# Efficient Polytelluride Anchoring for Ultralong-Life Potassium Storage: Combined Physical Barrier and Chemisorption in Nanogrid-in-Nanofiber

**DOI:** 10.1007/s40820-023-01318-9

**Published:** 2024-01-08

**Authors:** Qinghua Li, Dandan Yu, Jian Peng, Wei Zhang, Jianlian Huang, Zhixin Liang, Junling Wang, Zeyu Lin, Shiyun Xiong, Jiazhao Wang, Shaoming Huang

**Affiliations:** 1 Guangzhou Key Laboratory of Low-Dimensional Materials and Energy Storage Devices, School of Materials and Energy, Guangdong University of Technology, Guangzhou, 510006 People’s Republic of China; 2 College of Materials and Chemistry, China Jiliang University, Hangzhou, 310018 People’s Republic of China; 3 Institute for Superconducting and Electronic Materials, Australian Institute for Innovative Materials, University of Wollongong, Innovation Campus, Squires Way, North Wollongong, NSW 2522 Australia

**Keywords:** Polytelluride dissolution, Nanogrid-in-nanofiber structure, Physicochemical adsorption, Reaction mechanism, Ultralong-life potassium storage

## Abstract

Metal tellurides (MTes) are highly attractive as promising anodes for high-performance potassium-ion batteries. The capacity attenuation of most reported MTe anodes is attributed to their poor electrical conductivity and large volume variation. The evolution mechanisms, dissolution properties, and corresponding manipulation strategies of intermediates (K-polytellurides, K-pTe_*x*_) are rarely mentioned. Herein, we propose a novel structural engineering strategy to confine ultrafine CoTe_2_ nanodots in hierarchical nanogrid-in-nanofiber carbon substrates (CoTe_2_@NC@NSPCNFs) for smooth immobilization of K-pTe_*x*_ and highly reversible conversion of CoTe_2_ by manipulating the intense electrochemical reaction process. Various in situ/ex situ techniques and density functional theory calculations have been performed to clarify the formation, transformation, and dissolution of K-pTe_*x*_ (K_5_Te_3_ and K_2_Te), as well as verifying the robust physical barrier and the strong chemisorption of K_5_Te_3_ and K_2_Te on S, N co-doped dual-type carbon substrates. Additionally, the hierarchical nanogrid-in-nanofiber nanostructure increases the chemical anchoring sites for K-pTe_*x*_, provides sufficient volume buffer space, and constructs highly interconnected conductive microcircuits, further propelling the battery reaction to new heights (3500 cycles at 2.0 A g^−1^). Furthermore, the full cells further demonstrate the potential for practical applications. This work provides new insights into manipulating K-pTe_*x*_ in the design of ultralong-cycling MTe anodes for advanced PIBs.

**Supplementary Information:**

The online version contains supplementary material available at 10.1007/s40820-023-01318-9.

## Highlights

- The hierarchical nanogrid-in-nanofiber-structured dual-type carbon-confined CoTe_2_ nanodots (CoTe_2_@NC@NSPCNFs) were synthesized via facile templates and an electrospinning approach.
- Hierarchical nanogrid-in-nanofiber structure effectively suppresses the volume change of CoTe2 and the shuttle of potassium polytelluride (K-pTe_*x*_) through robust physical restraint and strong chemisorption.
- CoTe_2_@NC@NSPCNFs hybrid achieves ultralong lifespan potassium-storage performance over 3500 cycles, and the deep mechanisms underlying the evolution, dissolution, and shuttle of K-pTe_*x*_ have been clearly revealed.

**Supplementary Information:**

The online version contains supplementary material available at 10.1007/s40820-023-01318-9.

## Graphical abstract

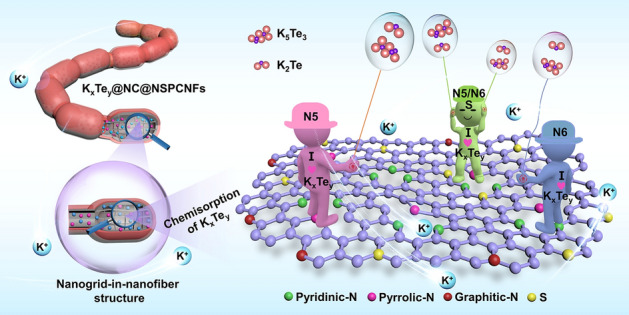

## Introduction

Potassium-ion batteries (PIBs) have been regarded as the most promising alternative to lithium-ion batteries (LIBs) in grid energy storage systems, owing to their earth-abundant resources, decently energy density, low electronegativity, and analogous physicochemical/electrochemical characteristics [1–5]. Nevertheless, serious volume changes and sluggish kinetics will occur during the potassiation/depotassiation processes due to the large K-ion radius (1.38 Å), resulting in nanostructure pulverization, low-rate capability, and rapid capacity fading of electrode materials [2, 6].

To date, tremendous efforts have been devoted to exploring appropriate anode materials for high-energy PIBs, including carbonaceous materials (graphite, soft carbon, and hard carbon) [7], alloy-type materials (Sb, Bi, and Sn) [8], metal oxides (BiSbO_4_, K_*x*_V_2_O_5_, and Fe_2_VO_4_) [9, 10], and metal chalcogenides (MCs, C=S, Se, Te) [11], and so on. Among the MCs, metal sulfides/selenides have drawn great attention as promising anodes for PIBs owing to their high theoretical specific capacity and widespread availability [12]. Obviously, the electrochemical properties of MCs are profoundly influenced by the corresponding anions (S, Se, and Te) in terms of their conductivity, volume effects, reaction kinetics, and solubility of the intermediates [12, 13]. It is well known that metal sulfides/selenides are subjected to large volume change effects, poor electrical conductivity (5.0 × 10^−28^ S m^−1^ for S, 1.0 × 10^−3^ S m^−1^ for Se), slow reaction kinetics, and an uncontrollable shuttle effect, resulting in severe capacity attenuation [12]. In contrast, metal tellurides (MTes) exhibit obvious good kinetics and stability due to the excellent physicochemical properties of the anionic element (semimetal Te), such as lower electronegativity, high electronic conductivity (2.0 × 10^2^ S m^−1^), and excellent theoretical gravimetric/volumetric capacity (419 mAh g^−1^/2621 mAh cm^−3^) [12, 14]. In particular, MTes (such as CoTe, FeTe_2_, *h*-CoTe_2_, MoTe_2_, Sb_2_Te_3_, and Bi_2_Te_3_) [12, 15–17] electrodes exhibit excellent rate capability because of their inspiring electrical conductivity, weak metal-Te bond energy, and high density (Table S1). Nevertheless, the previously reported MTes (CoTe_2_, MoTe_2_, FeTe_2_, Bi_2_Te_3_) [15–18] usually exhibit severe capacity attenuation, particularly during the initial stage of cycling, which is usually caused by severe volume effects and sluggish kinetics. Accordingly, traditional strategies have been employed, including designing special nanostructures to shorten ion transport distances and increase the availability of active substances [18], cladding a carbon-recombination layer to suppress volume effects and improve electrical conductivity [19], and constructing internal electric fields through the vacancy defect engineering strategy to enhance ionic conductivity [12]. Despite the successes achieved through the optimization strategies described above, there are still significant challenges to the long cycle life of MTe anodes. Furthermore, it can be concluded that there are still potential mechanisms that could compromise the potassium-storage performance of MTe anodes, but the detailed mechanism has not yet been revealed. Our recent work has revealed the dissociation and shuttling behavior of the K-pTe_*x*_ in MTes-based conversion and alloy-type anodes [20]. Meanwhile, the ability of nitrogen-doped carbon to effectively anchor K-pTe_*x*_ via chemisorption has been probed. Despite these promising results, the study of MTes materials is still in its infancy and there are still some aspects that need to be explored scientifically. For example, it is necessary to search for more rapid and sustained chemical anchoring strategies to address the diverse K-pTe_*x*_ and to construct appropriate porous structures that can be explored to accommodate the release of volumetric stresses arising during the conversion reaction. These research avenues of research hold the potential to further enhance the performance and understanding of MTes materials for advanced PIBs.

Herein, we have successfully synthesized a hierarchical nanogrid-in-nanofiber-structured dual-type carbon-confined CoTe_2_ composites (CoTe_2_@NC@NSPCNFs, where NSPCNFs stands for the outer N, S co-doped porous carbon nanofiber armor layer and NC represents the inner N-doped carbon grid) via a facile template, perfusion technique, and electrospinning approach, and we have further explored their potential in inhibiting the dissolution and shuttle effect of K-pTe_*x*_. Various in situ characterizations revealed the detailed “intercalation-conversion” mechanism of the CoTe_2_@NC@NSPCNFs anode and the formation/multi-step transformation processes of K-pTe_*x*_ (from K_*x*_CoTe_2_ to K_5_Te_3_, and eventually reduced to K_2_Te). Impressively, the in situ ultraviolet–visible (UV–Vis) absorption spectra and density functional theory (DFT) calculation results further prove that the dual-type carbon substrates offer vigorous physical confinement of K-pTe_*x*_, while pyridinic-N (N6) and pyrrolic-N (N5) doped carbon sites simultaneously exhibit an amazing chemical anchoring effect on K_5_Te_3_ and K_2_Te, especially in the case of N, S co-doping. Furthermore, the nanogrid-in-nanofiber structure enriches the chemical anchoring sites for K-pTe_*x*_, constructs sufficient volumetric-stress release space, and forms highly interconnected microcircuits to accelerate the electron/ion transport kinetics. Combining all the above features, the as-prepared CoTe_2_@NC@NSPCNFs electrode exhibits a high specific capacity (428.9 mAh g^−1^/1084.1 mAh cm^−3^ at 0.05 A g^−1^), superior cycling stability (194.5 mAh g^−1^/529.0 mAh cm^−3^ after 2000 cycles at 1.0 A g^−1^ with a capacity fading of only 0.005% per cycle, and 118.5 mAh g^−1^/322.3 mAh cm^−3^ after 3500 cycles at 2.0 A g^−1^ with ultraslow capacity decay rate of 0.02% per cycle). Finally, the CoTe_2_@NC@NSPCNFs anode-based dual-ion batteries and potassium-ion full cells exhibit outstanding cyclability and practicality. This work may provide promising inspiration for manipulating K-pTe_*x*_ to construct long-life PIBs.

## Experimental Section

### Synthesis of KCl–PVP–TU–Co NFs

Potassium chloride (KCl)–polyvinyl pyrrolidone (PVP)–thiourea (TU)–cobalt chloride (Co) nanofibers (NFs) were fabricated via typical electrospinning from a precursor solution that included KCl, PVP, TU, Co, and deionized water. During the electrospinning process, the precursor solution was injected into a plastic syringe (23^#^ needle) with a flow rate of 0.12 mm min^−1^. The voltage of 18 kV was applied with a distance of 15 cm between the nozzle tip and the collector. Finally, the collected nanofibers (KCl–PVP–TU–Co NFs) were dried at 80 °C under vacuum for further application.

### Synthesis of CoTe_2_@NC@NSPCNFs and CoTe_2_@NSPCNFs

The as-obtained KCl–PVP–TU–Co NFs were stabilized at 250 °C for 4 h in air with a heating rate of 2 °C min^−1^ and then carbonized at 450 °C for 2 h with a ramping rate of 2 °C min^−1^ under an argon atmosphere, with the product denoted as KCl–Co precursor@NSPCNFs. After that, the KCl–Co precursor@NSPCNFs were washed with deionized water to remove the KCl template and dried at 80 °C under vacuum for 12 h (with the product denoted as Co precursor@NSPCNFs). Subsequently, the Co precursor@NSPCNFs were fitted with PAN through immersion in a PAN solution (0.4 g PAN was dissolved in 10 mL of DMF) under vacuum for 8 h, and the product was collected by centrifugation and dried in a vacuum oven at 80 °C for 12 h. Finally, the above product and Te powder (the upstream side of the tube furnace) were placed separately on two sides of quartz boats in a mass ratio of 1:2 and further annealed at 600 °C for 2 h under an H_2_/Ar atmosphere with a ramping rate of 2 °C min^−1^, and the product was denoted as CoTe_2_@NC@NSPCNFs. For comparison, Co precursor@NSPCNFs were sintered with Te powder using the method of CoTe_2_@NC@NSPCNFs, and the final product was denoted as CoTe_2_@NSPCNFs.

### Synthesis of Pure CoTe_2_

Firstly, 0.5 mmol Na_2_TeO_3_ and 1.0 mmol Co(AC)_2_ were ultrasonically dispersed into a mixed solution (40 mL diethylenetriamine and 20 mL deionized water), and the above solution was stirred vigorously for 30 min. Secondly, the above solution was transferred into a Teflon-lined stainless-steel autoclave and heated at 180 °C for 16 h. Finally, the product (CoTe_2_) was collected by centrifugation, washed with deionized water and ethanol, and dried at 80 °C under vacuum for further application.

### Synthesis of S/N-Doped Pyrolytic Carbon Nanofibers (NC@NSPCNFs)

To obtain the NC@NSPCNFs from CoTe_2_@NC@NSPCNFs, Co precursor@NCNFs were dispersed in HCl solution (2 M), etched for 24 h, then washed with deionized water and ethanol (pH ≈ 7), and further dried in a vacuum oven at 80 °C for 12 h. Subsequently, the above product was fitted with PAN and carbonized without Te powder using the above method for CoTe_2_@NC@NSPCNFs. Finally, the as-obtained product was collected for further application and denoted as NC@NSPCNFs.

The detailed material characterizations, electrochemical measurements, and DFT simulations are provided in Supplementary Information.

## Results and Discussion

### Synthesis and Characterizations of the CoTe_2_-based Composites

**Fig. 1:**
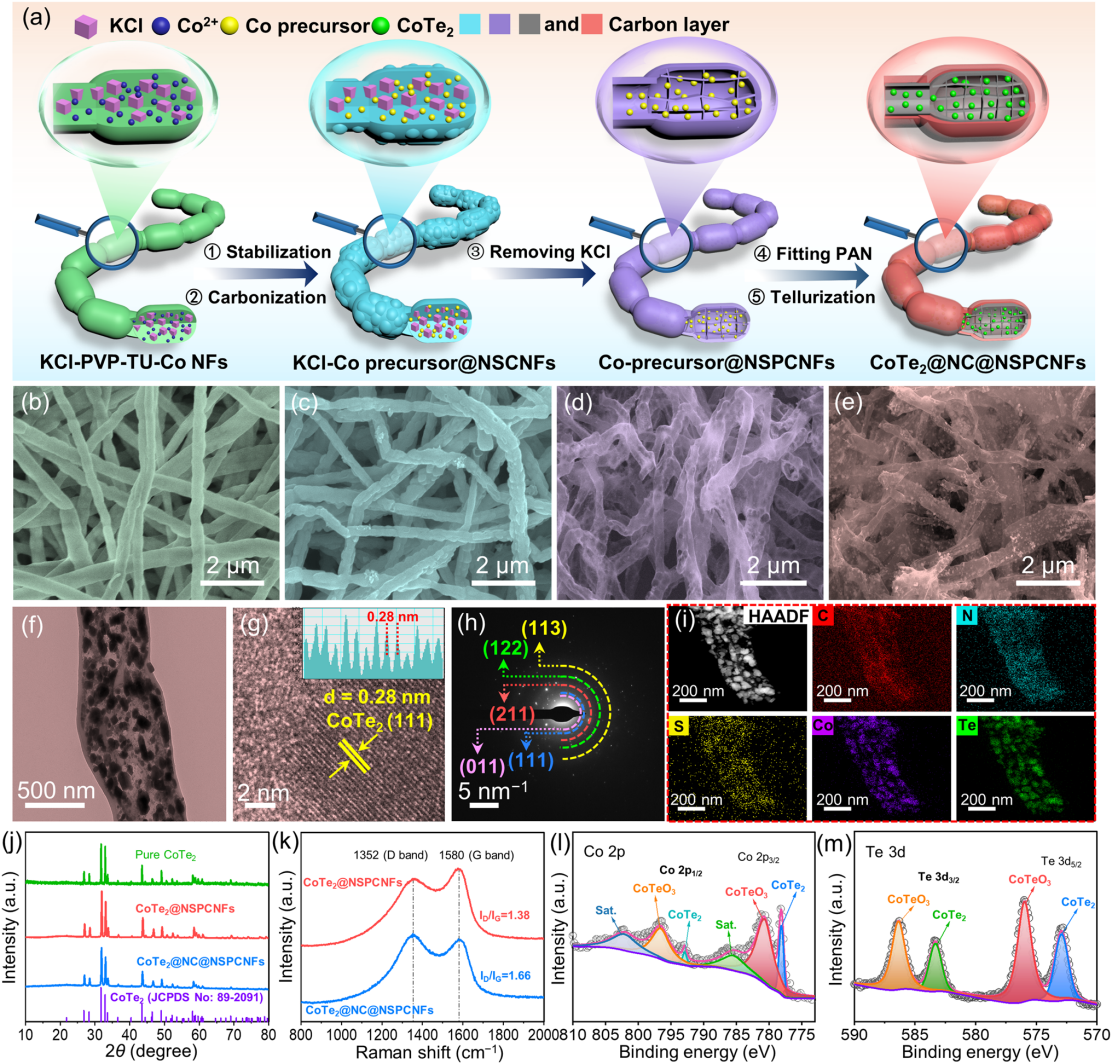
**a** Schematic illustration of the synthesis of CoTe_2_@NC@NSPCNFs. SEM images of **b** KCl–PVP–TU–Co NFs, **c** KCl–Co precursor@NPCNFs, **d** Co precursor@NPCNFs, and **e** CoTe_2_@NC@NSPCNFs. **f** TEM image, **g** HRTEM image with the inset showing the lattice spacing, **h** SAED pattern, and **i** elemental mapping images of CoTe_2_@NC@NSPCNFs. **j** XRD patterns of CoTe_2_@NC@NSPCNFs, CoTe_2_@NSPCNFs, and pure CoTe_2_. **k** Raman spectra of CoTe_2_@NC@NSPCNFs and CoTe_2_@NSPCNFs. XPS high-resolution spectra of **l** Co 2*p* and **m** Te 3*d* in CoTe_2_@NC@NSPCNFs

The hierarchical nanogrid-in-nanofiber-structured dual-type carbon-confined CoTe_2_ nanodots (CoTe_2_@NC@NSPCNFs) were first fabricated via facile templates, the perfusion technique, and an electrospinning approach, as presented in Fig. 1a. Here, for the first time, potassium chloride (KCl) crystals have been dispersed in hydrogels as s sacrificial template for electrospinning because of their high solubility, excellent thermal stability, and easy removal by washing with water. In addition, the KCl sacrificial template and adequate polyacrylonitrile (PAN) perfusion are the keys to the formation of the nanogrid inside the nanofibers, which inhibits the uncontrolled growth of CoTe_2_ particles during calcination, constructs large spatial mitigation to reduce the volume effect, and stabilizes the electrode structure during repeated charge/discharge processes.

The evolution of the morphology from KCl–polyvinyl pyrrolidone–thiourea–cobalt chloride nanofibers (KCl–PVP–TU–Co NFs), KCl–Co precursor@NPCNFs, and Co precursor @NSPCNFs to CoTe_2_@NC@NSPCNFs was recorded by scanning electron microscopy (SEM) and transmission electron microscopy (TEM). As displayed in Fig. 1b–e, the samples of KCl–PVP–Co NFs and KCl–Co precursor@NPCNFs embedded with KCl crystals exhibit a uniform bamboo-like fiber structure, while the Co precursor@NSPCNFs and CoTe_2_@NC@NSPCNFs show a translucent hollow porous fiber morphology. Notably, CoTe_2_ nanoparticles are uniformly embedded in the internal porous nanofibers, which is attributed to the confinement effect of the nanogrid structure (Fig. 1e, f). The internal gridded structure and the outer dense carbon armor layer are also clearly observed, as shown in Figs. 1f and S1. For comparison, the carbon-confined CoTe_2_ (CoTe_2_@NSPCNFs) composite shows significant precipitation and agglomeration of CoTe_2_ particles due to the loss of the inner PAN-derived carbon confinement, white pure CoTe_2_ exhibits a pronounced lump (Figs. S2a–c and S3). The high-resolution TEM (HRTEM) images of CoTe_2_@NC@NSPCNFs and CoTe_2_@NSPCNFs (Figs. 1g and S2d) show lattice spacings of 0.28 nm corresponding to the (111) planes of CoTe_2_ (JCPDS no. 89-2091) [21]. The presence of CoTe_2_ nanocrystals in CoTe_2_@NC@NSPCNFs was further confirmed by the selected area electron diffraction pattern (SAED) (Fig. 1h). In addition, C, N, S, Co, and Te elements are homogeneously distributed throughout the porous carbon skeleton of CoTe_2_@NC@NSPCNFs, while the Co and Te elements in CoTe_2_@NSPCNFs exhibit clear aggregation (Figs. 1i and S2e, f).

The X-ray diffraction (XRD) patterns of CoTe_2_@NC@NSPCNFs, CoTe_2_@NSPCNFs, and pure CoTe_2_ in Fig. 1j exhibit a series of diffraction peaks, which match well with the orthorhombic CoTe_2_ phase (*Pnn2* space group, JCPDS no. 89–2091) [14, 21]. The intensity ratio (*I*_D_/*I*_G_) of the D band (1352 cm^−1^) to the G band (1580 cm^−1^) of CoTe_2_@NC@NSPCNFs and CoTe_2_@NSPCNFs was 1.66 and 1.38, respectively. The high *I*_D_/*I*_G_ value implies a highly disordered carbon matrix in CoTe_2_@NC@NSPCNFs, which facilitates the capture of additional K^+^ and improves the K-ion storage kinetics (Fig. 1k) [14, 22–24]. The CoTe_2_ weight ratio in CoTe_2_@NC@NSPCNFs was calculated to be 65.9 wt% according to the thermogravimetric analysis (TGA) result and the formation of Co_2_Te_3_O_8_ as the oxidation product (as exhibited in Fig. S4). Moreover, the nanogrid-in-nanofiber-structured CoTe_2_@NC@NSPCNFs present a high specific surface area (136.3 m^2^ g^−1^) and hierarchical porous structure (with pore diameters ranging from 1.7 to 5.0 nm) (Fig. S5a), which not only constructs the volume variation space for CoTe_2_ but also provides a network for high electron conductivity. In addition, the electronic states and chemical-bond types of CoTe_2_@NC@NSPCNFs were characterized by X-ray photoelectron spectroscopy (XPS). The survey spectrum verifies the presence of C, N, S, Co, and Te elements (Fig. S5b). Moreover, the nitrogen and sulfur contents were 7.5 and 1.3 atom% based on XPS results, respectively (Table S2). As presented in Fig. S5c, the high-resolution spectrum of C1s can be assigned to the C–C (248.8 eV), C–O/C–N/C–S (286.4 eV), and O–C = O/N–C = O (289.2 eV) bonds, respectively [22]. The S 2*p* spectrum exhibits two peaks at 163.5 and 164.7 eV corresponding to S 2*p*_3/2_ (S–S) and S 2*p*_1/2_ (C–S) (Fig. S5d) [25, 26]. In addition, the binding energy of the peaks in the Co 2*p* spectrum (Fig. 1l), located at 778.1 and 792.9 eV, can be attributed to the Co 2*p*_1/2_ and Co 2*p*_3/2_ orbitals of CoTe_2_, respectively [12, 14, 27]. Moreover, the peaks at around 780.7 and 796.6 eV are indexed to the surface oxidation of CoTe_2_ (CoTeO_3_) [12, 27]. The peaks at 572.9 and 583.3 eV in the high-resolution Te 3*d* spectrum of CoTe_2_@NC@NSPCNFs (Fig. 1m) are attributed to the Te 3*d*_5/2_ and Te 3*d*_3/2_ orbitals of CoTe_2_, respectively, while the peaks located at 576.0 and 586.4 eV are assigned to the Te–O bonding structure (in the cobalt tellurite on the surface of CoTe_2_) [12, 14, 19, 27]. Meanwhile, the N 1*s* spectrum of CoTe_2_@NC@NSPCNFs (Fig. S5e) can be split into three peaks (398.2, 400.1, and 401.3 eV), corresponding to N6, N5, and graphitic-N (NQ) [24, 28, 29]. Notably, the relative atomic content of N6, N5, and NQ is 43.5%, 43.2%, and 13.2%, respectively (Fig. S6 and Table S3). It is well known that N6 and N5 can increase charge storage sites, improve electrical conductivity, facilitate ion/electron transfer, and further provide various active sites for interfacial adsorption in the capacitive process [29–31].

### Electrochemical Performance of CoTe_2_@NC@NSPCNFs

**Fig. 2:**
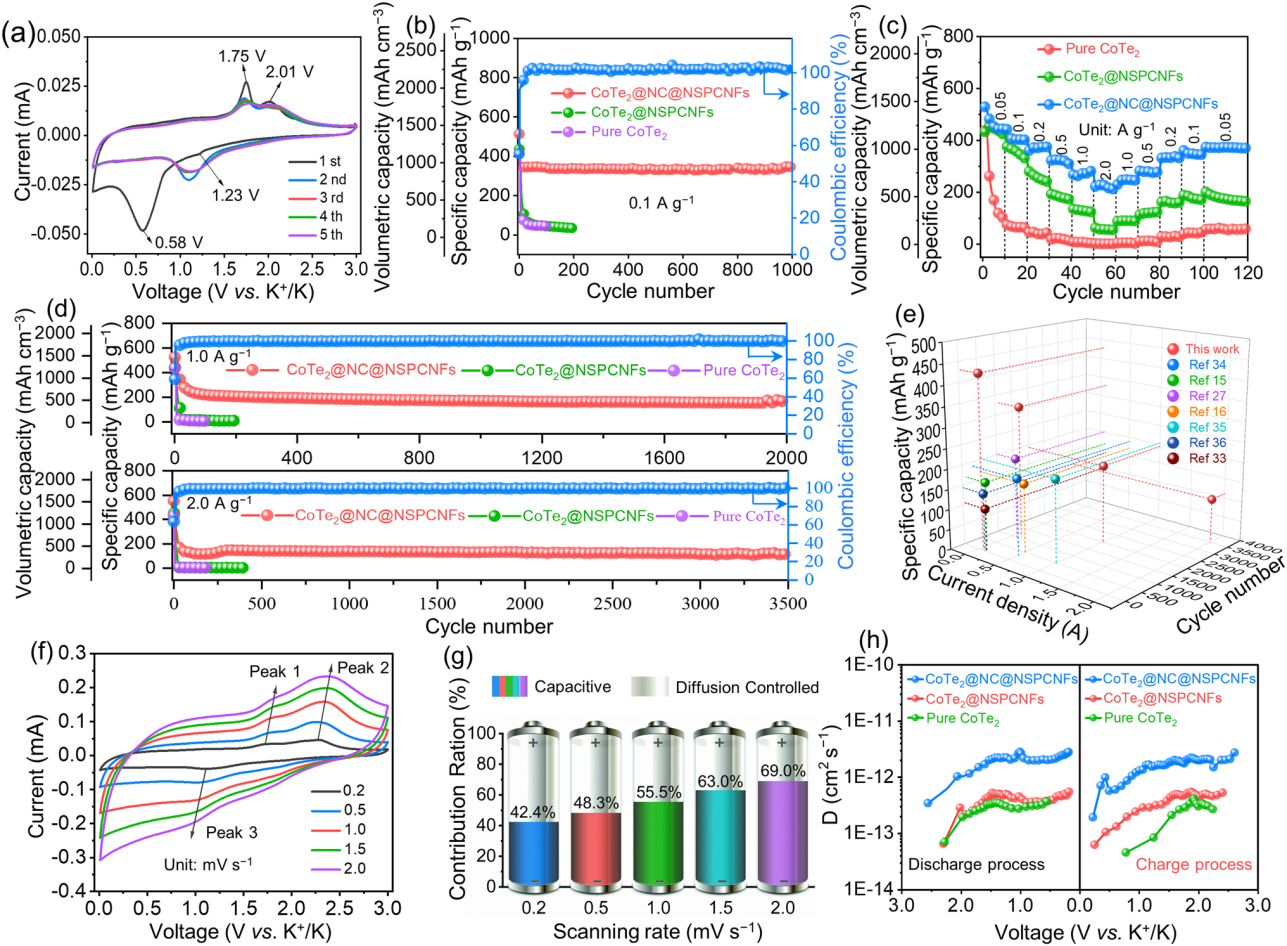
**a** CV curves of the CoTe_2_@NC@NSPCNFs electrode at 0.1 mV s^−1^. **b** Cycling performance of the CoTe_2_@NC@NSPCNFs electrode at 0.1 A g^−1^. **c** Rate performance of the CoTe_2_@NC@NSPCNFs, CoTe_2_@NSPCNFs, and pure CoTe_2_ electrodes. **d** Cycling performance of the CoTe_2_@NC@NSPCNFs electrode at 1.0 and 2.0 A g^−1^. **e** Comparison of the potassium-storage performance of the CoTe_2_@NC@NSPCNFs anode with the previously reported Te-based anodes. **f** CV curves at various scan rates and **g** contribution ratios of the capacitive-controlled capacity of the CoTe_2_@NC@NSPCNFs electrode. **h** K^+^ diffusion coefficients (*D*_K+_) of the CoTe_2_@NC@NSPCNFs, CoTe_2_@NSPCNFs, and pure CoTe_2_ electrodes calculated from GITT curves

The potassium-storage performance of the as-prepared electrodes was assessed in coin-type half cells with K metal as the counter/reference electrode. Figure 2a shows the initial five cyclic voltammetry (CV) curves of the CoTe_2_@NC@NSPCNFs electrode, which were collected in the voltage range of 0.01–3.0 V at the scan rate of 0.1 mV s^−1^. In the first cathodic scan, the peak at 1.23 V is assigned to the formation of K_*x*_CoTe_2_ (the intercalation of K^+^ into the CoTe_2_ nanocrystals) and the stable solid-electrolyte interphase (SEI) film [12, 14, 27]. Meanwhile, the reduction peak that appeared at 0.58 V is indexed to the conversion from CoTe_2_ to metal Co and K-pTe_*x*_ [12, 32]. Moreover, two obvious anodic peaks, located at 1.75 and 2.01 V in the following anodic scan, correspond to the stepwise conversion reaction from K-pTe_*x*_ to CoTe_2_ [12, 32]. After that, the CV curves almost overlap during cycling from the second to the fifth cycles, demonstrating highly reversible electrochemical behavior. The galvanostatic charge/discharge profiles (Fig. S7a) also show a stable potassium-storage process and an initial Coulombic efficiency of 55.6%. Notably, the CoTe_2_@NC@NSPCNFs electrode delivers a significant specific capacity of 428.9 mAh g^−1^/1084.1 mAh cm^−3^ after 50 cycles at 0.05 A g^−1^ (Fig. S7b). Furthermore, the CoTe_2_@NC@NSPCNFs electrode maintains a reversible capacity of 335.9 mAh g^−1^/923.6 mAh cm^−3^ after 1000 cycles at 0.1 A g^−1^ with a capacity fading of only 0.005% per cycle (Fig. 2b), while the CoTe_2_@NSPCNFs and pure CoTe_2_ display a low capacity after 200 and 100 cycles, respectively. Impressively, the CoTe_2_@NC@NSPCNFs electrode achieves higher reversible capacities of 512.5, 406.1, 372.2, 325.9, 263.1, and 228.2 mAh g^−1^ (corresponding to the volumetric capacities of 1394.0, 1104.6, 1012.4, 886.4, 715.6, and 620.7 mAh cm^−3^) than those of CoTe_2_@NSPCNFs and pure CoTe_2_ electrodes under various current densities from 0.5 to 2.0 A g^−1^, respectively (Figs. 2c and S8). Importantly, the CoTe_2_@NC@NSPCNFs electrode maintains an inspiring specific capacity of 194.5 mAh g^−1^/529.0 mAh cm^−3^ after 2000 cycles at 1.0 A g^−1^ and 118.5 mAh g^−1^/322.3 mAh cm^−3^ after 3500 cycles at 2.0 A g^−1^ (Fig. 2d), where the capacity contribution from the S, N co-doped pyrolytic carbon nanofibers (NC@NSPCNFs) was only 8.7% at 1.0 A g^−1^ and 2.6% at 2.0 A g^−1^, respectively (Fig. S9 and Table S4). As a comparison, the capacities of CoTe_2_@NSPCNFs and pure CoTe_2_ electrodes decay rapidly and almost reach zero at 1.0 and 2.0 A g^−1^. To the best of our knowledge, the remarkable cyclability of the CoTe_2_@NC@NSPCNFs anode is the longest recorded cycling lifespan among the previously reported Te-based anode materials in PIBs (Fig. 2e and Table S5) [15, 16, 27, 33–36]. The outstanding performance of CoTe_2_@NC@NSPCNFs indicates the successful design of its hierarchical nanogrid-in-nanofiber structure.

To gain a deep insight into the ultra-stable K-storage performance of the CoTe_2_@NC@NSPCNFs electrode, the electrochemical impedance spectroscopy (EIS), measurement of the redox pseudo-capacitance contribution, and galvanostatic intermittent titration technique (GITT) were performed to evaluate the electrode kinetics. As displayed in Fig. S10a–c and Tables S6–S8, the results from the EIS curves and the corresponding fitted resistances show that the charge transfer resistance (*R*_ct_) of the CoTe_2_@NC@NSPCNFs electrode decreases and stabilizes after 50 cycles, while those of the CoTe_2_@NSPCNFs and pure CoTe_2_ electrodes decrease after five cycles and then significantly increase after 50 cycles, which indicates the superior structural stability of the nanogrid-in-nanofiber-structured CoTe_2_@NC@NSPCNFs anode (the morphology changes of the CoTe_2_@NC@NSPCNFs, CoTe_2_@NSPCNFs, and pure CoTe_2_ electrode before and after 50 cycles are exhibited in Fig. S10d–i, and the detailed discussion is shown in Supplementary Information). Notably, the similar cathodic/anodic peaks in the CV curves (Fig. 2f) shift to the opposite direction with increasing scan rate. The corresponding values of the *b* parameter for the cathodic and anodic peaks are 0.70, 0.71, and 0.70, respectively (Fig. S11a), implying a surface capacitance-dominated process for the CoTe_2_@NC@NSPCNFs anode. Furthermore, the ratio of the capacitive contribution of the CoTe_2_@NC@NSPCNFs anode is 42.4, 48.3, 55.5, 63.0, and 69.0% at the scan rate of 0.2, 0.5, 1.0, 1.5, and 2.0 mV s^−1^, respectively (Fig. S11b). Such a high ratio of capacitive contribution to the diffusion-controlled contribution can be attributed to the sufficient active surface and the abundance of N, S co-doping sites in the hierarchical nanogrid-in-nanofiber structure of CoTe_2_@NC@NSPCNFs, which is beneficial to the improvement of rate capability and reaction kinetics. In addition, as shown in Figs. 2h and S12, the CoTe_2_@NC@NSPCNFs electrode has a higher K^+^ diffusion coefficient (*D*_K+_) value (3.47 × 10^−13^–2.73 × 10^−12^ cm^2^ s^−1^) than those of CoTe_2_@NSPCNFs (6.55 × 10^−14^–5.50 × 10^−13^ cm^2^ s^−1^) and pure CoTe_2_ electrodes (7.05 × 10^−14^–3.69 × 10^−13^ cm^2^ s^−1^) during the discharge process. Meanwhile, the CoTe_2_@NC@NSPCNFs electrode also displays a higher* D*_K+_ value(1.94 × 10^−13^–2.74 × 10^−12^ cm^2^ s^−1^) than that of CoTe_2_@NSPCNFs (6.29 × 10^−14^–5.32 × 10^−13^ cm^2^ s^−1^) and pure CoTe_2_ electrodes (4.58 × 10^−14^–4.16 × 10^−13^ cm^2^ s^−1^) during charge process (the calculation details given in Supplementary Information). Overall, the CoTe_2_@NC@NSPCNFs electrode demonstrated fast K^+^ migration throughout the entire potassiation/depotassiation processes, indicating that the nanogrid-in-nanofiber structure favors the acceleration of K^+^ diffusion kinetics, thereby promoting the rate capability [14, 22, 37].

### Electrochemical Mechanism Analysis of CoTe_2_@NC@NSPCNFs

**Fig. 3:**
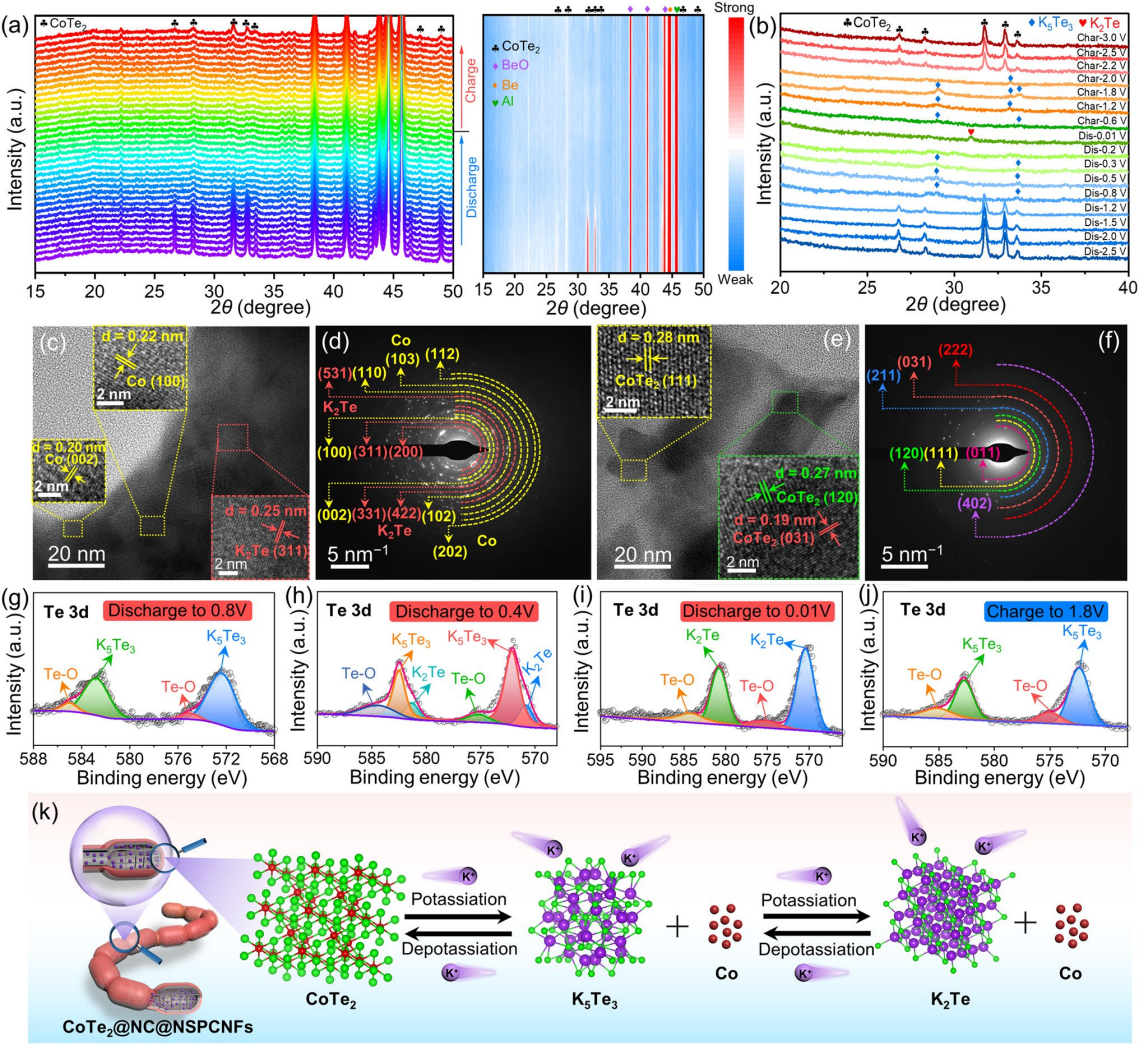
**a** In situ XRD patterns and the corresponding contour plot and **b** *ex situ* XRD patterns of CoTe_2_@NC@NSPCNFs electrode during the first cycle at 0.02 A g^−1^. **c**, **e** HRTEM images and **d**, **f** SAED patterns of the CoTe_2_@NC@NSPCNFs electrode after discharge to 0.01 V and charge to 3.0 V at 0.02 A g^−1^, respectively. High-resolution Te 3d spectra of CoTe_2_@NC@NSPCNFs electrodes at different discharged and charged states: **g** discharge to 0.8 V, **h** discharge to 0.4 V, **i** discharge to 0.01 V, and **j** charge to 3.0 V. **k** Schematic illustration of the potassium-storage mechanism of the CoTe_2_@NC@NSPCNFs electrode

To reveal the potassium-storage mechanism of the CoTe_2_@NC@NSPCNFs electrode, in situ and ex situ XRD were employed. At the initial state of open-circuit voltage (OCV), the characteristic peaks of the CoTe_2_@NC@NSPCNFs anode located at 26.8°, 28.3°, 31.7°, 32.9°, 46.4°, and 49.1° are well matched with the (011), (101), (111), (120), (022), and (031) planes of orthorhombic CoTe_2_ (Fig. 3a) [21, 38]. In addition, the unchanged peaks situated at 38.5°, 41.2°, 43.9°, 44.7°, and 45.8° can be ascribed to BeO, Al foil, and pure Be, respectively. During the potassiation process (OCV to 1.23 V), the peaks of CoTe_2_ become weak and shift slightly toward a lower angle (Fig. S13), demonstrating the intercalation of K^+^ into the CoTe_2_ nanocrystal to form K_*x*_CoTe_2_. As the sustained potential decreases to 0.01 V, the peak intensity of CoTe_2_ gradually vanishes, elucidating the conversion processes from CoTe_2_ to Co metal and K-pTe_*x*_. Notably, there are no obvious peaks of Co metal and K-pTe_*x*_ in the deep discharging state due to the attenuating effect of the in situ XRD mold window. Subsequently, the ex situ XRD results clearly elucidate the gradual transformation of CoTe_2_ into K_5_Te_3_ (~ 0.8 V, JCPDS no. 79-1056) and K_2_Te (less than 0.2 V, JCPDS no. 77-2154) during the potassiation process, and completely reversible transformation from K_2_Te to K_5_Te_3_ (~ 0.6 V) and then CoTe_2_ (~ 2.0 V) takes place when the voltage returns to 3.0 V (Fig. 3b). Correspondingly, the discharge (Co and K_5_Te_3_) and charge products (CoTe_2_) are also confirmed by the HRTEM and SAED. Specifically, a series of lattice fringes with spacings of 0.20, 0.22, 0.25, and 0.33 nm appear, corresponding to the (002) and (100) planes of Co metal (JCPDS no. 97-005-2935), the (311) plane of K_2_Te, and the (321) plane of K_5_Te_3_, respectively (Fig. 3c). On charging to 3.0 V, some lattice fringes with a spacing of 0.19, 0.27, and 0.28 nm emerge (Fig. 3e), which are indexed to the (031), (120), and (111) planes of CoTe_2_, respectively. These results were well supported by the results of SAED (Fig. 3d, f), suggesting that the potassiation/depotassiation processes of the CoTe_2_@NC@NPCNF anode are highly reversible. Excellent reversibility also benefits from the robust nanogrid-in-nanofiber structure of CoTe_2_@NC@NPCNF electrode and homogeneous distribution of C, N, S, Co, and Te elements during the initial potassiation/depotassiation processes (Figs. S14 and S15).

Ex situ XPS analysis (Figs. 3g–j and S16) was further conducted to clarify the composition evolution of the CoTe_2_@NC@NSPCNFs anode during cycling. After discharge to 0.8 V (Fig. 3g), the peaks in the Te 3*d* spectrum located at 572.3 (Te 3*d*_5/2_) and 582.7 eV (Te 3*d*_3/2_) can be assigned to K_5_Te_3_, while the peaks at 575.0 and 585.1 eV are attributed to the surface oxidation (Te–O band) [33]. Obviously, the characteristic peaks of K_5_Te_3_ gradually weaken (at 0.4 V) and disappear (at 0.01 V) along with the appearance of K_2_Te at 570.5 eV (Fig. 3h–i). Simultaneously, the characteristic peaks of Co metal gradually becomes strong during the discharge process (Fig. S16a –c). Upon recharging to 1.8 V (Figs. 3j and S16d), the characteristic peaks of K_5_Te_3_ reappear, accompanied by the disappearance of the K_2_Te and the weakening of the Co metal, further indicating that the Co metal is a redox center for the reversible conversion process (Fig. S16d) [39]. Interestingly, the binding energy of K 2*p* spectra at different voltage states of the CoTe_2_@NC@NPCNF anode, located at 292.5 and 294.8 eV, is attributed to the formation of K_2_CO_3_ contained in the SEI layer (Fig. S16e–h) [25]. Subsequently, the results of in situ EIS measurements (Fig. S17) demonstrated that the charge transfer resistance (*R*_ct_) initially increases and then decreases during the discharge process. The increase in *R*_ct_ is ascribed to the intercalation behavior to produce K_*x*_CoTe_2_ with low conductivity, while the decrease in *R*_ct_ is attributed to the converted production of Co metal with good conductivity and the formation of SEI film. During the charging process, the *R*_ct_ value displays high reversibility, indicating superior structural stability of the CoTe_2_@NC@NSPCNFs anode [21]. In addition, the regularly evolving and reversible D and G bands and corresponding *I*_D_/*I*_G_ values once again demonstrate the stability of the hierarchical nanogrid-in-nanofiber structure during the intercalation/deintercalation of K^+^ (Fig. S18) [40–42]. Based on the above results (in situ XRD and EIS, ex situ XRD/XPS/TEM) and the CV curves, the reaction mechanism of the CoTe_2_@NC@NSPCNFs anode (Fig. 3k) can be divided into six stages and described as follows:

*Stage I* CoTe_2_ + *x*K^+^  + *x*e^–^ → K_*x*_CoTe_2_ (Intercalation reaction).

*Stage II* 3K_*x*_CoTe_2_ + (10 − 3*x*)K^+^  + (10 − 3*x*)e^–^ → 3Co + 2K_5_Te_3_ (Conversion reaction).

*Stage III* K_5_Te_3_ + K^+^  + e^–^ → 3K_2_Te (Conversion reaction).

*Stage IV* 3K_2_Te → K_5_Te_3_ + K^+^  + e^–^ (Reverse conversion reaction).

*Stage V* 2K_5_Te_3_ + 3Co → 3K_*x*_CoTe_2_ + (10 − 3*x*)K^+^  + (10 − 3*x*)e^–^ (Reverse conversion reaction).

*Stage VI* K_*x*_CoTe_2_ → CoTe_2_ + *x*K^+^  + *x*e^–^ (Deintercalation reaction).

### Dissolution Behavior and DFT Calculations of CoTe_2_@NC@NSPCNFs

**Fig. 4:**
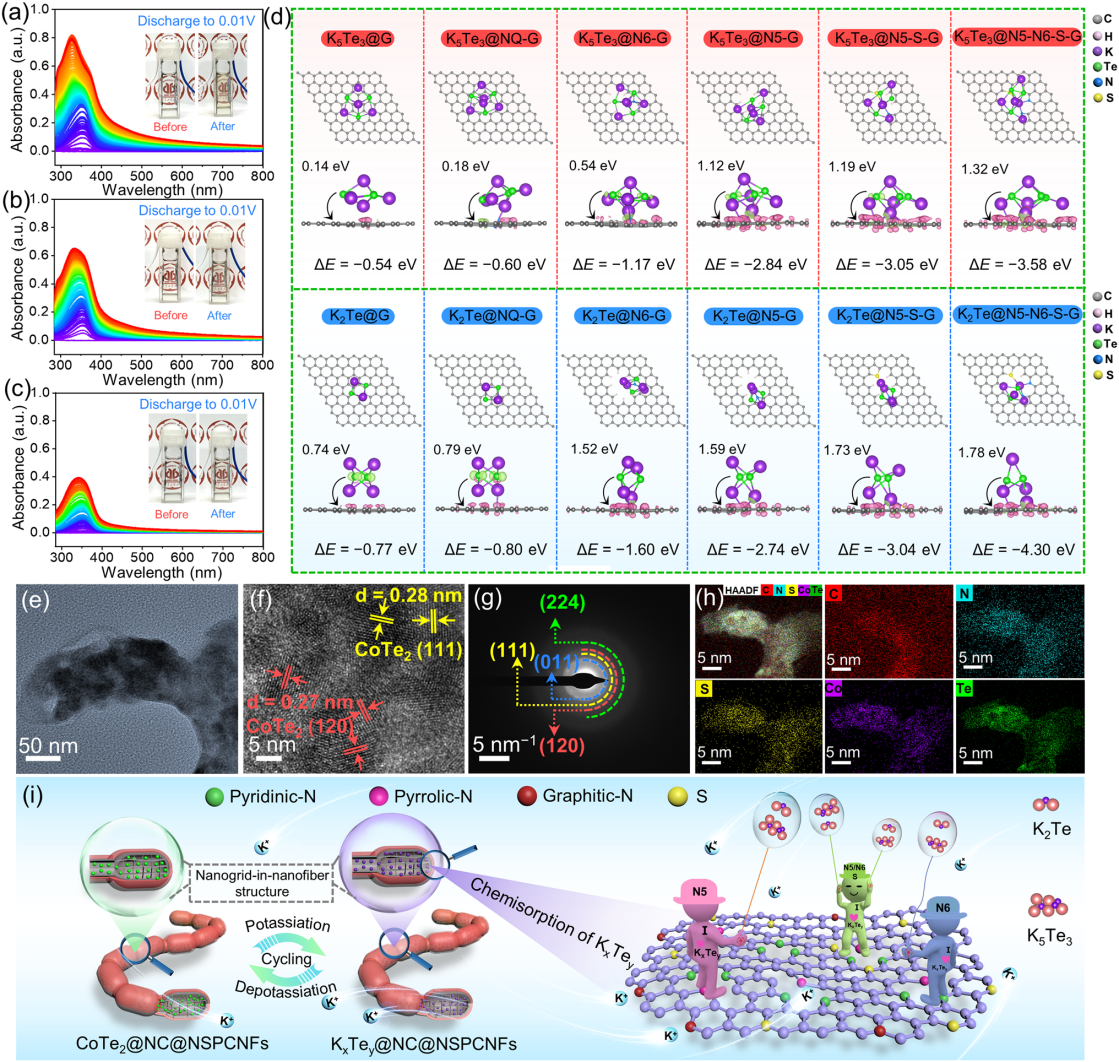
In situ UV–Vis absorption spectra of **a** CoTe_2_@NC@NSPCNFs, **b** CoTe_2_@NSPCNFs, and **c** pure CoTe_2_ electrode. Insets to **a**, **b**, and **c** are the electrolytic cells for the collection of *in situ* UV–Vis absorption spectra before and after the initial fully discharged state, respectively. **d** Top and side view illustrations of simulations of K_5_Te_3_ and K_2_Te adsorbed on different graphene substrates and side views of the corresponding electron density differences. Pink and light green areas represent electron accumulation and depletion, respectively. **e** TEM and **f** HRTEM images, **g** corresponding SAED pattern, and **h** EDS mapping images of the CoTe_2_@NC@NSPCNFs electrode after 1000 cycles at 1.0 A g^−1^. **i** Schematic illustration of the CoTe_2_@NC@NSPCNFs anode during the repeated potassiation and depotassiation processes

To elucidate the dissolution of K-pTe_*x*_ and explore the mechanism behind the inhibition of dissolution during the potassium-storage process, in situ UV–Vis absorption spectra and DFT calculations were employed. The in situ UV–Vis absorption spectra of an electrolytic cell were collected based on three different anodes (CoTe_2_@NC@NSPCNFs, CoTe_2_@NSPCNFs, and pure CoTe_2_) with the 3 M KFSI/DME electrolyte during the initial discharge process at 0.05 A g^−1^ (Figs. S19 and S20). Obviously, the absorption peak intensity of the DME solution containing the pure CoTe_2_ electrode rapidly increases with the continuous potassiation (Fig. 4a), representing the rapid dissolution of K-pTe_*x*_, which is responsible for the rapid capacity decay. In addition, the continuous shift of the absorption peak toward lower wavelengths (from 350 to 327 nm) corresponds to the gradual evolution from K_5_Te_3_ to K_2_Te [43]. Subsequently, the UV–Vis peak intensity of the DME solution containing the CoTe_2_@NSPCNFs electrode (Fig. 4b) greatly decreases, indicating a significant anchoring effect between the heteroatoms (N and S atoms) and K-pTe_*x*_ after the doping of N and S atoms into the hierarchical nanogrid-in-nanofiber. Interestingly, the corresponding absorption peak does not obviously shift, even after discharge to 0.01 V, indicating that N and S atoms have a strong chemical anchoring capability toward K_5_Te_3_. Benefiting from the synergistic effect of chemical anchoring of N/S atoms and the physical confinement of the nanogrid-in-nanofiber carbon structure, the CoTe_2_@NC@NSPCNFs electrode (Fig. 4c) exhibits an amazing capability to inhibit the dissolution of K-pTe_*x*_, which can be confirmed by the UV–Vis peak intensity and photographs of electrolytic cells (Fig. S20).

To further illustrate the anchoring effect of the N, S co-doped hierarchical nanogrid-in-nanofiber on K-pTe_x_ (K_5_Te_3_ and K_2_Te) in the CoTe_2_@NC@NSPCNFs electrodes, different graphene substrates (graphene (G), graphitic-N (NQ-G), pyridinic-N (N6-G), pyrrolic-N (N5-G), pyrrolic-N, S co-doped (N5-S-G), and pyridinic-N/pyrrolic-N, S co-doped graphene (N5-N6-S-G)) were employed (with detailed calculations described in Supporting Information). As shown in Figs. 4d and S21, the adsorption energies (∆*E*) of K_5_Te_3_ on different carbon substrates including G, NQ-G, N6-G, N5-G, N5-S-G, and N5-N6-S-G are − 0.54, − 0.60, − 1.17, − 2.84, − 3.05, and − 3.58 eV, respectively. Meanwhile, the adsorption energies of K_2_Te on G, NQ-G, N6-G, N5-G, N5-S-G, and N5-N6-S-G also reach − 0.77, − 0.80, − 1.60, − 2.74, − 3.04, and − 4.3 eV, respectively. Impressively, we have verified that N5 has stronger adsorption energy toward K_5_Te_3_ and K_2_Te than N6, NQ, and G; meanwhile N, S co-doped graphene (N5-S-G and N5-N6-S-G) has the strongest chemical adsorption capability, which is essential for achieving ultra-stable cycling. Charge density difference plots (Figs. 4d and S22) indicate that charges are transferred from K_5_Te_3_ and K_2_Te molecules to carbon substrates, and heteroatoms can accelerate electron transfer and achieve fast reaction kinetics. In particular, the electron density is inclined to accumulate more around the N–S sites, further confirming the stronger adsorption between K-pTe_*x*_ and the N5, N6, and S co-doped carbon skeleton. Astonishingly, the dual anchoring mechanism incorporating carbon physical barriers and heteroatomic chemisorption inhibits the loss of active material from the CoTe_2_@NC@NSPCNFs electrode and improves the cycling stability. In addition, the CoTe_2_ lattice fringes and crystal planes can still be observed on the CoTe_2_@NC@NSPCNFs electrode, even after 1000 cycles (Fig. 4e–g). Moreover, the C, N, S, Co, and Te elements are uniformly distributed in the carbon skeleton (Fig. 4h), implying the excellent stability of the hierarchical nanogrid-in-nanofiber structure during the repeated potassiation/depotassiation. Based on the above results, the physical barrier of dual-type carbon skeletons and chemisorption on heteroatomic sites, especially S/N co-doping, can effectively inhibit the dissolution and shuttle effects of K_5_Te_3_/K_2_Te. More importantly, the hierarchical nanogrid-in-nanofiber structure provides sufficient volume buffer space for the conversion reaction and constructs an abundance of chemical anchoring sites and robust physical confinement layers for K_5_Te_3_/K_2_Te, which is the key to obtaining ultra-stable potassium storage in CoTe_2_@NC@NSPCNFs anode (Fig. 4i).

### Electrochemical Performance of the Full Cell

**Fig. 5:**
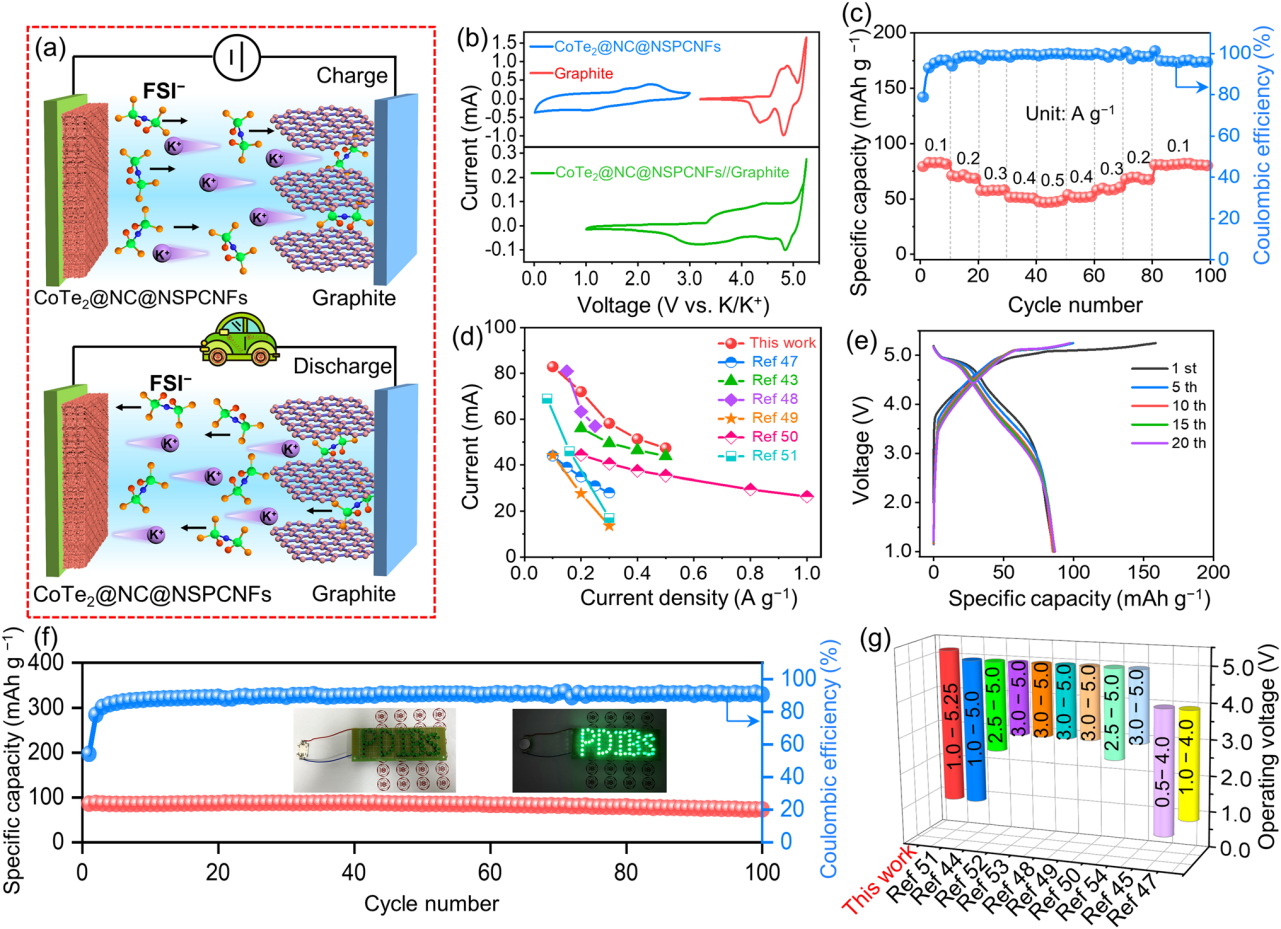
**a** Schematic illustration of the CoTe_2_@NC@NSPCNFs//graphite PDIB. **b** CV curves of the CoTe_2_@NC@NSPCNFs anode and graphite cathode in half cells, and the CoTe_2_@NC@NSPCNFs//graphite PDIB. Electrochemical performance of the CoTe_2_@NC@NSPCNFs//graphite PDIB: **c** rate capability, **d** comparison of rate performance between the previously reported PDIB and this work, **e** charge/discharge profiles at 0.1 A g^−1^, **f** cycling performance at 0.1 A g^−1^ (inset: photograph of LED arrays was powered by one CoTe_2_@NC@NSPCNFs//graphite PDIB), and **g** the operating voltage compared with those of the previously reported PDIBs

To further assess the practical applications, potassium-based dual-ion batteries (PDIBs) were assembled by using graphite as cathode and CoTe_2_@NC@NSPCNFs as anode with 5 M KFSI/ethylene carbonate (EC)/dimethyl carbonate (DMC) electrolyte. The charge/discharge states of CoTe_2_@NC@NSPCNFs//graphite PDIBs are schematically illustrated in Fig. 5a. During the charging process, K^+^ cations in the electrolyte migrate to the anode and react with CoTe_2_; meanwhile, FSI^−^ anions in the electrolyte move to the cathode and intercalate into the graphitic layer. Subsequently, K^+^ cations and FSI^−^ anions return to the electrolyte during the discharged process [43–45]. It should be noted that 5 M KFSI/EC/DMC electrolyte was used in PDIBs instead of 3 M KFSI/DME electrolyte due to its excellent oxidation resistance at high operating voltages [43, 46]. The morphology of graphite and the cycling performance of the CoTe_2_@NC@NSPCNFs anode and graphite cathode in 5 M KFSI/EC/DMC were investigated, and the results are displayed in Figs. S23, S24, and S25, respectively. The CV curves of two half cells (the CoTe_2_@NC@NSPCNFs anode and graphite cathode) and a full cell (CoTe_2_@NC@NSPCNFs//graphite) are illustrated in Fig. 5b. For the CoTe_2_@NC@NSPCNFs anode, the operating voltage window is from 0.01 to 0.3 V, corresponding to a conversion-type mechanism. But for graphite cathode, there are obvious redox peaks within the operating voltage window of 3.2–5.25 V, suggesting an intercalation-type process. The CoTe_2_@NC@NSPCNFs anodes were coupled with graphite cathodes to construct PDIBs with an operating potential from 1.0 to 5.25 V. The capacity of CoTe_2_@NC@NSPCNFs//graphite PDIBs (Figs. 5c and S26) is 82.5, 71.6, 57.7, 51.2, and 47.4 mAh g^−1^ (based on the mass of graphite in the cathode) at the current density of 0.1, 0.2, 0.3, 0.4, and 0.5 A g^−1^, respectively, suggesting the superior rate capability compared with those of the previously reported PDIBs (Fig. 5d) [43, 47–51]. The charge/discharge curves of the CoTe_2_@NC@NSPCNFs//graphite PIBs at the 1st, 5th, 10th, 15th, and 20th cycles are displayed in Fig. 5e. It is found that the charge/discharge curves overlap well, demonstrating the good cycling stability of the PIBs. Furthermore, the CoTe_2_@NC@NSPCNFs//graphite PDIBs exhibit a high specific capacity of 73.2 mAh g^−1^ after 100 cycles at 0.1 A g^−1^ (Fig. 5f). Moreover, the CoTe_2_@NC@NSPCNFs//graphite PDIB can powder the light-emitting diode (LED) array with the label of “PDIBs”. Impressively, the wide operating voltage (1.0–5.25 V) of the CoTe_2_@NC@NSPCNFs//graphite PDIBs compared with other PDIBs further indicates their great potential for practical applications (Fig. 5g and Table S9) [44, 45, 47–54]. In addition, a K-ion full cell was assembled coupled with potassium Prussian blue (KPB) cathodes to further evaluate the feasibility of the CoTe_2_@NC@NSPCNFs anode. The CoTe_2_@NC@NSPCNFs//KPB full cell also exhibits the reversible capacity of 93.8 and 81.9 mAh g^–1^ at 0.1 and 0.5 A g^–1^ after 100 and 200 cycles, respectively, suggesting the great potential of the CoTe_2_@NC@NSPCNFs anode for the high-performance K-ion full cells (Figs. S27, S28, S29, the detailed information shown in Supplementary Information).

## Conclusions

In summary, the hierarchical nanogrid-in-nanofiber-structured dual-type carbon-confined CoTe_2_@NC@NSPCNFs anode was fabricated to reveal the intrinsic mechanism of K-pTe_*x*_ evolution and dissolution during K-storage in MTe anodes and explore possible inhibition strategies. The underlying gradual conversion mechanism, severe dissolution, and shuttle effect of the K-pTe_*x*_ are investigated in detail by in situ XRD/UV–Vis and ex situ XPS/TEM analysis. Benefiting from the synergistic contribution of the hierarchical nanogrid-in-nanofiber structure, which provides a rich volume buffer space, robust physical confinement, and vigorous chemical adsorption energy at S, N co-doping sites, the as-obtained CoTe_2_@NC@NSPCNFs composite delivers an ultralong-cycling performance and excellent rate capability (194.5 mAh g^−1^/529.0 mAh cm^−3^ after 2000 cycles at 1.0 A g^−1^, and 118.5 mAh g^−1^/322.3 mAh cm^−3^ after 3500 cycles at 2.0 A g^−1^ with ultraslow capacity decay rate of 0.02% per cycle). In addition, the CoTe_2_@NC@NSPCNFs//graphite PDIBs deliver a high specific capacity of 73.2 mAh g^−1^ after 100 cycles, and the CoTe_2_@NC@NSPCNFs//KPB full cell exhibits the reversible capacity of 81.9 mAh g^–1^ after 200 cycles, which further demonstrates the potential of MTe anode for practical applications. The fundamental understanding of the mechanism and rational nanostructural design provide a promising direction for suppressing the shuttle effect of the soluble K-pTe_*x*_ in MTe anode for PIBs.

## Supplementary Information

Below is the link to the electronic supplementary material.

Supplementary file1 (PDF 3784 KB)
