# Supplementary material for: Efficient Polytelluride Anchoring for Ultralong-Life Potassium Storage: Combined Physical Barrier and Chemisorption in Nanogrid-in-Nanofiber
Source: Nanomicro Lett. 2024 Jan 8;16:77. doi: 10.1007/s40820-023-01318-9 (PMC10774503; doi:10.1007/s40820-023-01318-9)
Supplement: Supplementary file 1 — Supplementary file1 (PDF 3784 KB) [file 40820_2023_1318_MOESM1_ESM.pdf]

Supporting Information for

Efficient Polytelluride Anchoring for Ultralong-Life Potassium Storage: Combined Physical Barrier and Chemisorption in Nanogrid-in-Nanofiber

Qinghua Li[1], Dandan Yu[2], Jian Peng[3], Wei Zhang[1, *], Jianlian Huang[1], Zhixin Liang[1], Junling Wang[1], Zeyu Lin[1], Shiyun Xiong[1], Jiazhao Wang[3], Shaoming Huang[1, *]

[1] School of Materials and Energy, Guangzhou Key Laboratory of Low-Dimensional Materials and Energy Storage Devices, Guangdong University of Technology, Guangzhou 510006, P. R. China

[2] College of Materials and Chemistry, China Jiliang University, Hangzhou 310018, P. R. China

[3] Institute for Superconducting and Electronic Materials, Australian Institute for Innovative Materials, University of Wollongong, Innovation Campus, Squires Way, North Wollongong, NSW, 2522, Australia

* Corresponding authors. E-mail: zhwei@gdut.edu.cn (W. Zhang); smhuang@gdut.edu.cn (S. M. Huang)

S1 Materials Characterization

The phases and composition of samples were characterized by X-ray diffraction (XRD, SmartLab9KW with Cu-Kα radiation at 40 kV, λ = 1.541 Å), X-ray photoelectron spectroscopy (XPS, Escalab 250Xi), Raman spectroscopy (Renishaw InVia, 532 nm excitation wavelength) and energy-dispersive spectroscopy (EDS, FEI Talos F200S). The carbon content in $CoTe_2$@NC@NSPCNFs was examined by thermogravimetric analysis (TGA DSC 3+, Mettler Toledo) with a heating rate of 10 °C min^{-1} under air atmosphere. The microstructures and morphology of samples were investigated through scanning electron microscopy (SEM, Thermo Fisher Scientific Apreo C, USA) and transmission electron microscopy (TEM, Talos F200S, FEI, Thermo). The specific surface area and pore size distribution (PSD) were analyzed by the Brunauer-Emmett-Teller method (BET, Micromeritics ASAP2460) and the Barrett-Joyner-Halenda (BJH) method, respectively. Ultraviolet-visible (UV–vis) absorption spectra of the $CoTe_2$@NC@NSPCNFs, $CoTe_2$@NSPCNFs, and pure $CoTe_2$ anodes were collected during the initial fully discharged state at 0.02 A g^{-1} by using a UV-2600 spectrophotometer with a sampling interval of 0.2 nm.

S2 Electrochemical Measurements

The working electrodes were fabricated by mixing the active materials ($CoTe_2$@NC@NSPCNFs), CMC, SBR, and super P (in a weight ratio of 8: 0.5: 0.5: 1) with deionized water as the solvent. Then, the slurry was uniformly coated onto Cu foil and dried at 70 °C for 24 h under vacuum drying. The CR2032 coin-type half cells were assembled in an Ar-filled glove box with H_2O and O_2 contents less than 0.01 ppm. In the half-cells, K metal, glass fiber (Whatman), and 3 M KFSI in DME were used as the counter electrode, the separator, and the electrolyte, respectively. The mass loading of the active materials ($CoTe_2$@NC@NSPCNFs) was about 1.2 mg cm^{-2}. All the values of the specific capacity are based on the mass of the $CoTe_2$@NPCNFs@NC.

The graphite electrode was prepared using a weight ratio of 8:1:1 for natural graphite/super P/polyvinylidene fluoride (PVDF), using N-Methylpyrrolidone (NMP) as the solvent. After grinding for 30 min, the homogeneous slurry was coated onto aluminum (Al) foils and then vacuum dried at 70 ^{o}C for 12 h. The anion-storage performance of the graphite cathodes was characterized by assembling K-graphite potassium-based dual-ion batteries (PDIBs) with 5 M KFSI in EC/DMC (1:1, v/v) as the electrolyte, and the applied voltage window ranged from 3.2 to 5.25 V. The mass loading of active material in the graphite cathodes was around 1.5−2.0 mg cm^{-2}. It is worth noting that the $CoTe_2$@NC@NSPCNFs anodes were pre-potassiated before the PDIBs were assembled. The $CoTe_2$@NC@NSPCNFs anode was first cycled at 0.1 A g^{-1} for 5 cycles in half cells (counter electrodes: K metal), after which the half cells were discharged to 0.01 V. The $CoTe_2$@NC@NSPCNFs//graphite PDIBs were assembled by using the pre-potassiated $CoTe_2$@NC@NSPCNFs as the anode, graphite as the cathode, and 5 M KFSI in EC/DMC as the electrolyte within the voltage window of 1.0−5.25 V. The weight ratio of 3:1 (the active materials in the cathode and anode) was designed to keep the cathode-to-anode capacity ratio around 1.0-1.1.

The preparation of the KPB cathode is similar to that of the graphite cathode, but the active material (KPB), Super P, and PVDF binder are prepared in a mass ratio of 6:3:1. The performance of KPB cathode in half cells was evaluated by using 3 M KFSI in DME as the electrolyte and K metal as counter electrode under voltage window ranging from 2.0 to 4.0 V. To improve the cycling stability of $CoTe_2$@NC@NSPCNFs//KPB full cells, $CoTe_2$@NC@NSPCNFs electrodes were firstly pre-potassiated in half cells for 5 cycles at 0.1 A g^{-1} and further discharged to 0.01 V.

The $CoTe_2$@NC@NSPCNFs//KPB full cells were obtained with 2032 coin and pouch cells by utilizing $CoTe_2$@NC@NSPCNFs anode, KPB cathode, the electrolyte of 3 M KFSI in DME, and Whatman glass fibers serving as the separator within the voltage window of 0.1−3.6 V. The $CoTe_2$@NC@NSPCNFs anode was directly coupled with the KPB cathode at the mass ratio of 5:1 according to the capacity matching. All the above working and counter electrodes were cut into circle pieces with a diameter of 12 mm. Cyclic voltammetry (CV) measurements were performed on an Autolab instrument (PGSTAT 302) at a scan rate of 0.1 mV s^{-1}. Electrochemical impedance spectroscopy (EIS) was conducted on an electrochemical workstation (Autolab 302N) from 1×10^5 to 0.1 Hz. The galvanostatic charge/discharge tests were conducted on a Neware battery test system (CT-ZWJ-4'S-T-1U, Shenzhen, China). Galvanostatic intermittent titration technique (GITT) tests were conducted by discharging and charging the cells at 0.02 A g^{-1} for 30 min with a rest interval of 2.5 h in the range of 0.01 to 3.0 V. The volumetric specific capacity was calculated based on the tap density of $CoTe_2$@NC@NSPCNFs (2.72 g cm^{-3}). For the electrolytic cell of *in-situ* ultraviolet-visible (UV-vis) measurements, the working electrodes were prepared by mixing 80 wt% of the active materials (pure $CoTe_2$, $CoTe_2$@NSPCNFs, or $CoTe_2$@NC@NSPCNFs), 10 wt% of PVDF, and 10 wt% of super P with NMP as the solvent. The slurry was uniformly coated onto Al foil and dried at 70 ^{o}C for 24 h under a vacuum. K metal and 3 M KFSI in DME were used as the counter/reference electrode and the electrolyte, respectively.

S3 DFT Simulations

The Vienna Ab initio Simulation Package (VASP) was employed to perform all density functional theory (DFT) calculations within the generalized gradient approximation (GGA) using the Perdew-Burke-Ernzerhof (PBE)functional. The projected augmented wave (PAW) potentials were adopted to describe the ionic cores and take valence electrons into account using a plane-wave basis set with a kinetic energy cut-off of 400 eV. The DFT-D3 empirical correction method was employed to describe van der Waals interactions. All crystal structure relaxations

were conducted until the residual force acting on each atom was less than 0.05eV/Å. Additionally, the requirement for self-consistent calculation convergence was set at 10^{-5} eV. A Monkhorst-Pack k-grid of 1 × 1 × 1 was applied for all the calculations. The adsorption energy (E_a) was calculated by the equation: $E_a = E_{(slab + K_xTe_y)} - E_{(slab)} - E_{(K_xTe_y)}$, where $E_{(slab + K_xTe_y)}$ and $E_{(slab)}$ are the total energy of the surface slab with and without K_xTe_y, respectively, and $E_{(K_xTe_y)}$ is the total energy of the K_xTe_y molecule.

S4 Calculation Process for the $CoTe_2$ Content in $CoTe_2$@NC@NSPCNFs from TGA Analysis

The content of $CoTe_2$ in $CoTe_2$@NC@NSPCNFs was characterized by thermogravimetric analysis (TGA) and analyzed based on the weight loss from carbon combustion (Figure S4A) and the weight increment from the oxidation of elemental $CoTe_2$ to $Co_2Te_3O_8$ ($2CoTe_2 + 5O_2 + C \rightarrow Co_2Te_3O_8 + CO_2\uparrow + Te\uparrow$) (Fig. S4b) [S1, S2]. Therefore, the content of $CoTe_2$ in $CoTe_2$@NPCNFs@NC could be calculated by the following equation:

$$\mathrm{CoTe_2(wt\%)} = \mathrm{Co_2Te_3O_8(wt\%)} \times 2 \times \frac{M_{\mathrm{CoTe_2}}}{M_{Co_2Te_3O_8}} = \mathrm{Co_2Te_3O_8(wt\%)} \quad \text{(S1)}$$

where $M_{\mathrm{CoTe_2}}$ and $M_{Co_2Te_3O_8}$ are the molecular weights of $CoTe_2$ and $Co_2Te_3O_8$, respectively. The $CoTe_2$ content in the composite was calculated to be ~65.9 wt%.

S5 Calculation Process to Determine the Capacitance Effect and Pseudocapacitive Contribution

The capacitance effect can be determined from the curve, according to the relationship between measured peak currents (i), and scanning rates (v), as follows:

$$i = a\, v^b \quad \text{(S2)}$$

$$\log i = b \log v + \log a \quad \text{(S3)}$$

where a and b are the fitting parameters, and i and v represent the peak current and scan rate, respectively. The capacitive behavior could be estimated using the b value, which is the slope of the "log i *vs.* log v" plot [S2]. For diffusion-controlled behavior, the b-value approaches 0.5, while for a surface capacitance-dominated process, it is close to 1.0 [S3, S4]. Furthermore, the pseudocapacitive contribution can also be calculated by the following equation:

$$i = k_1\, v + k_2\, v^{1/2} \quad \text{(S4)}$$

where k_1v and $k_2v^{1/2}$ represent the pseudocapacitive contribution and the ionic diffusion contribution, respectively.

S6 Details of the Diffusion Coefficient (D_{K^+})

The galvanostatic intermittent titration technique (GITT) tests were performed by discharging or charging the cells for 30 min at 20 mA g^{-1}, followed by a 2.5 h relaxation in the range of 0.01 to 3.0 V. The diffusion coefficient can be worked out by solving Fick's second law according to Eq. (S5).

$$D = \frac{4}{\pi\tau}\left(\frac{m_B V_M}{M_B S}\right)^2 \left(\frac{\Delta E_S}{\Delta E_\tau}\right)^2 \left(\tau \ll \frac{L^2}{D}\right) \quad \text{(S5)}$$

where τ is the duration of the current impulse (s), and m_B, V_M, M_B, and S are the mass, the molar volume of the active material, the molar mass, and the area of the electrode, respectively. ΔE_S represents the quasi-thermodynamic equilibrium potential difference between the potentials before and after the current pulse. ΔE_τ is the potential difference during the current pulse [S5].

S7 Supplementary Figures and Tables

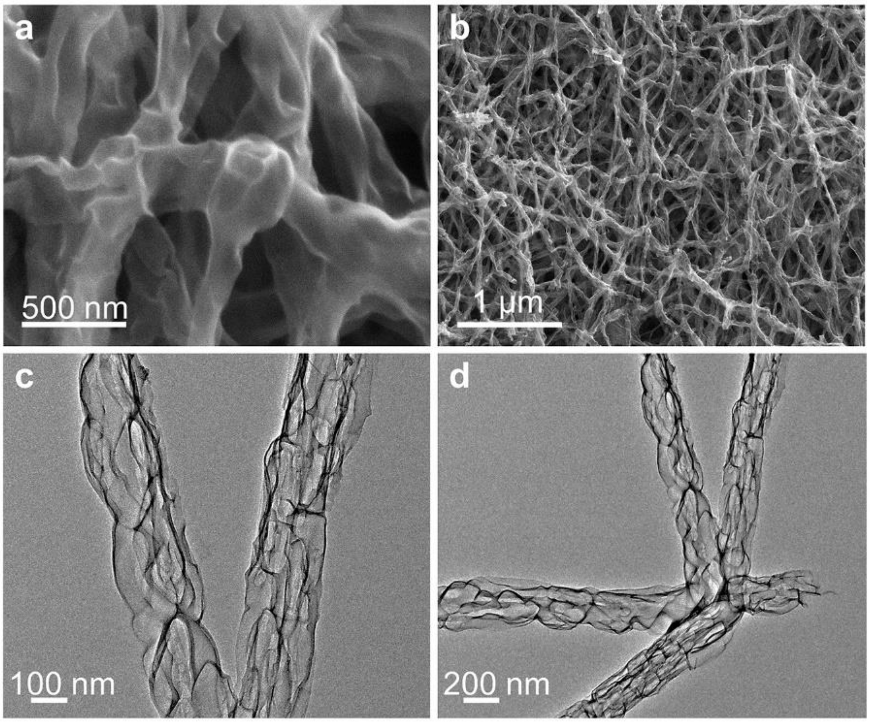

Fig. S1 (**a**, **b**) Scanning electron microscope (SEM) and (**c**, **d**) transmission electron microscopy (TEM) images of S, N co-doped pyrolytic carbon nanofibers (NC@NSPCNFs)

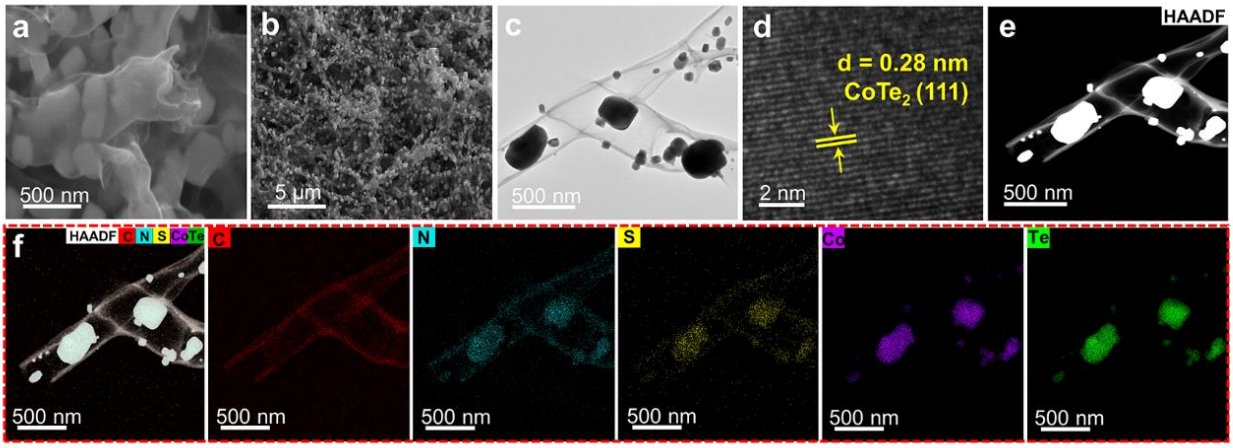

Fig. S2 (**a**, **b**) SEM, (**c**) TEM, (**d**) HRTEM, (**e**) HAADF, and (**f**) elemental mapping images of $CoTe_2$@NSPCNFs

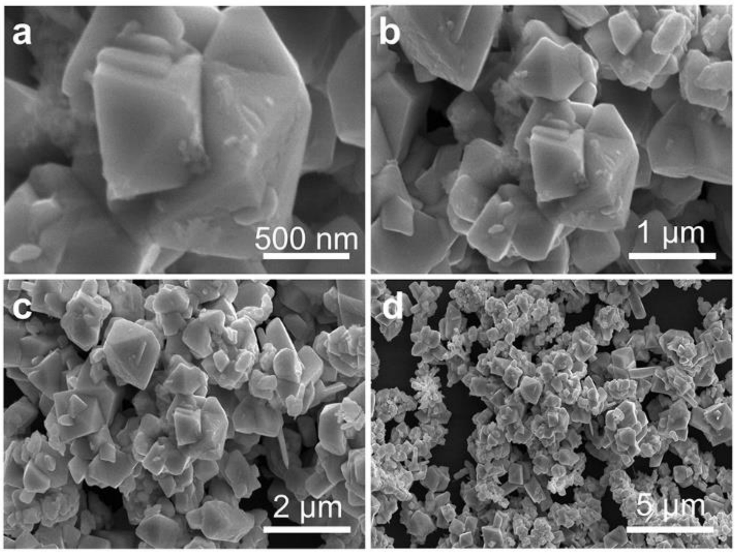

Fig. S3 SEM images of (**a-d**) pure $CoTe_2$. As displayed in Fig. S3, the morphology of pure $CoTe_2$ consists of blocks of various sizes

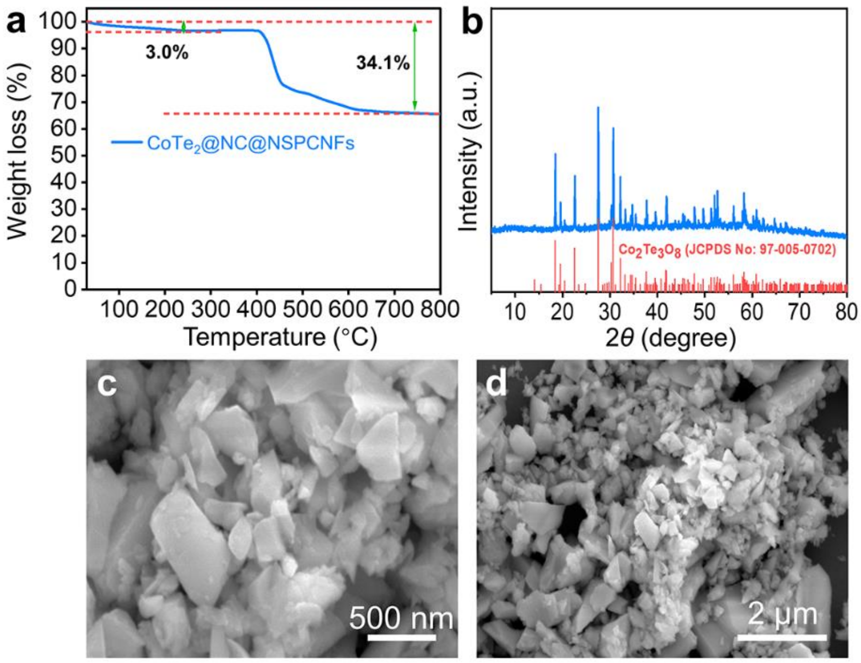

Fig. S4 (**a**) Thermogravimetric analysis (TGA) curve of $CoTe_2$@NC@NSPCNFs. (**b**) XRD patterns and (**c**, **d**) SEM images of $CoTe_2$@NC@NSPCNFs residue after TGA testing

As shown in Figs. S4a–b, according to the TGA result and oxidation product ($Co_2Te_3O_8$), the carbon content of $CoTe_2$@NC@NSPCNFs was calculated to be 34.1 wt%. It can also be seen in Figs. S4c-d that the morphology of $Co_2Te_3O_8$ is lumpy.

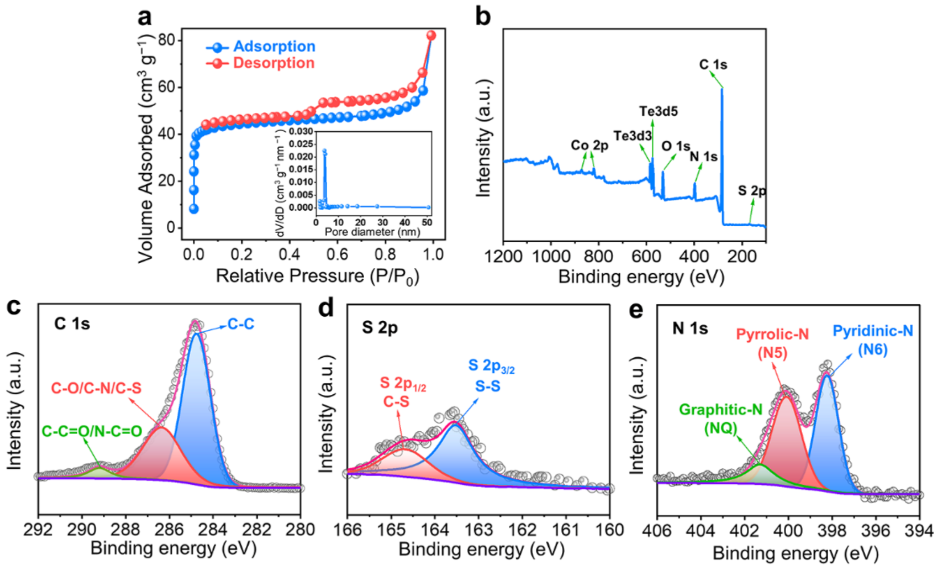

Fig. S5 (**a**) Nitrogen adsorption/desorption curves curve and pore size distribution (inset) of $CoTe_2$@NC@NSPCNFs. (**b**) XPS survey spectrum and high-resolution spectra of (**c**) C 1s, (**d**) S 2p, and (**e**) N 1s of $CoTe_2$@NC@NSPCNFs

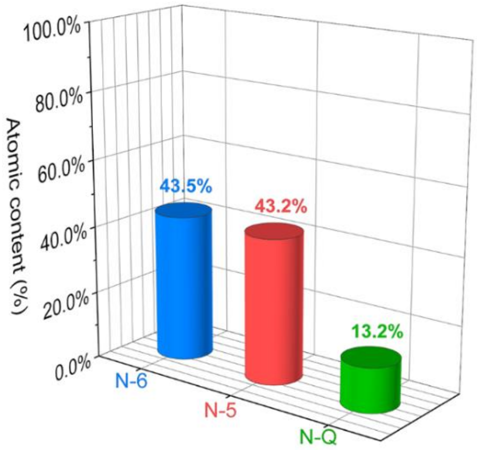

Fig. S6 Atomic contents of pyridinic-N (N-6), pyrrolic-N (N-5), and graphitic-N (N-Q) in $CoTe_2$@NC@NSPCNFs

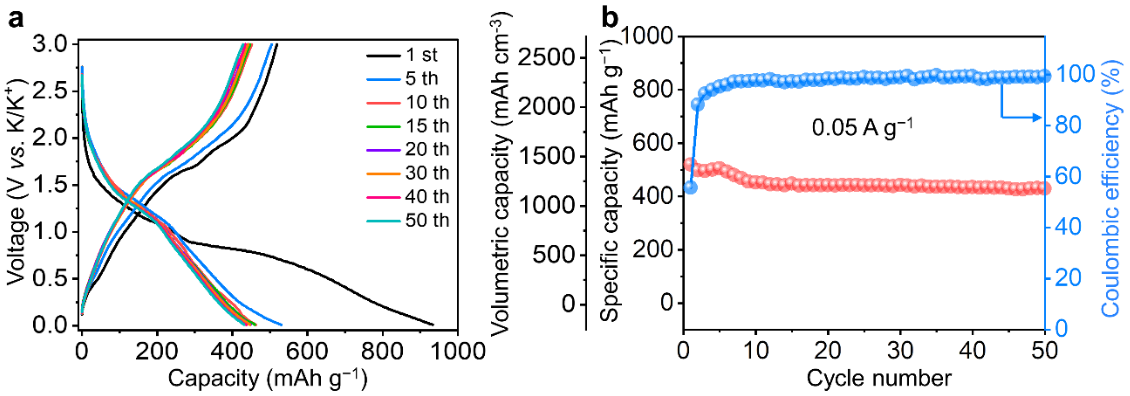

Fig. S7 (**a**) The galvanostatic charge-discharge profiles and (**b**) cycling performance of the $CoTe_2$@NC@NSPCNFs electrode at 0.05 A g^{-1}

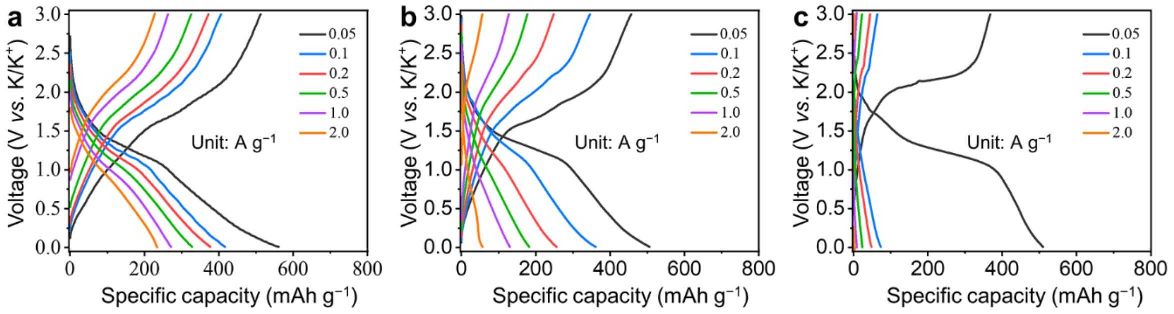

Fig. S8 Selected galvanostatic charge/discharge profiles of (**a**) the $CoTe_2$@NC@NSPCNFs, (**b**) $CoTe_2$@NSPCNFs, and (**c**) pure $CoTe_2$ electrodes at various current densities from 0.05 to 2.0 A g^{-1}

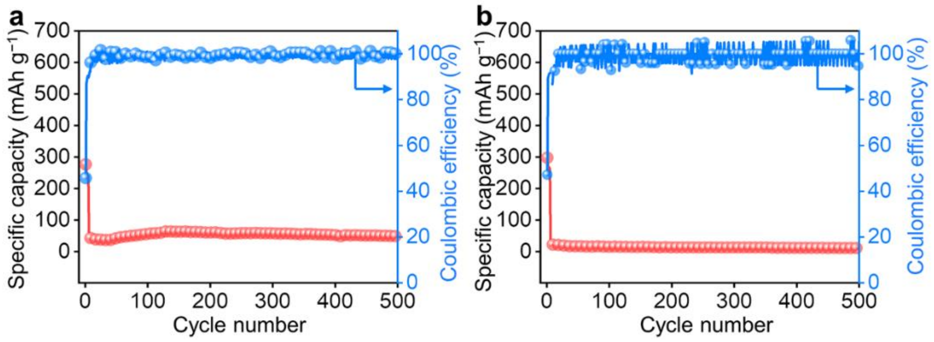

Fig. S9 Cycling performance of NC@NSPCNFs in the $CoTe_2$@NC@NSPCNFs electrode at a current density of (**a**) 1.0 and (**b**) 2.0 A g^{-1}

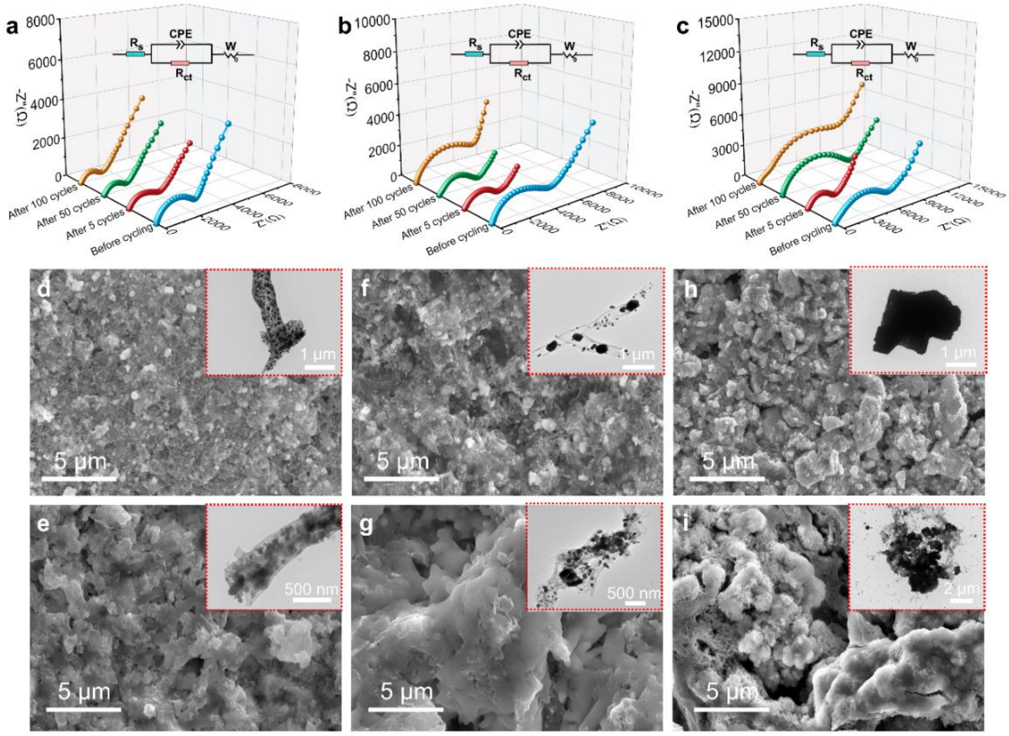

Fig. S10 EIS curves of (**a**) the $CoTe_2$@NC@NSPCNFs, (**b**) $CoTe_2$@NSPCNFs, and (**c**) pure $CoTe_2$ electrodes before and after different cycles at 0.1 A g^{-1}. The insets in (**a**, **b**, **c**) are their corresponding equivalent circuits. SEM images of (**d**, **e**) the $CoTe_2$@NC@NSPCNFs, (**f**, **g**)

$CoTe_2$@NSPCNFs, and (**h**, **i**) pure $CoTe_2$ electrodes before and after 50 cycles at 0.1 A g^{-1}, respectively. Insets are their corresponding TEM images

It can be clearly seen from Fig S10a that the charge transfer resistance (R_{ct}) of the $CoTe_2$@NC@NSPCNFs electrode decreases and stabilizes after 50 cycles, which is attributed to the robust nanogrid-in-nanofiber skeleton that can withstand the strain-induced by the volume change, well maintain the nanostructure, and facilitate the formation of a stable SEI layer during cycling (as exhibited in Fig S10d, e). However, the R_{ct} of the $CoTe_2$@NSPCNFs and pure $CoTe_2$ electrode decreases after 5 cycles and then significantly increases after 50 cycles (especially for the pure $CoTe_2$ electrode), which is ascribed to the huge volume change that occurs during the potassiation/depotassiation processes and eventually leads to severe pulverization of the structure, even the collapse of the conducting network after cycling (as displayed in Fig S10f-i).

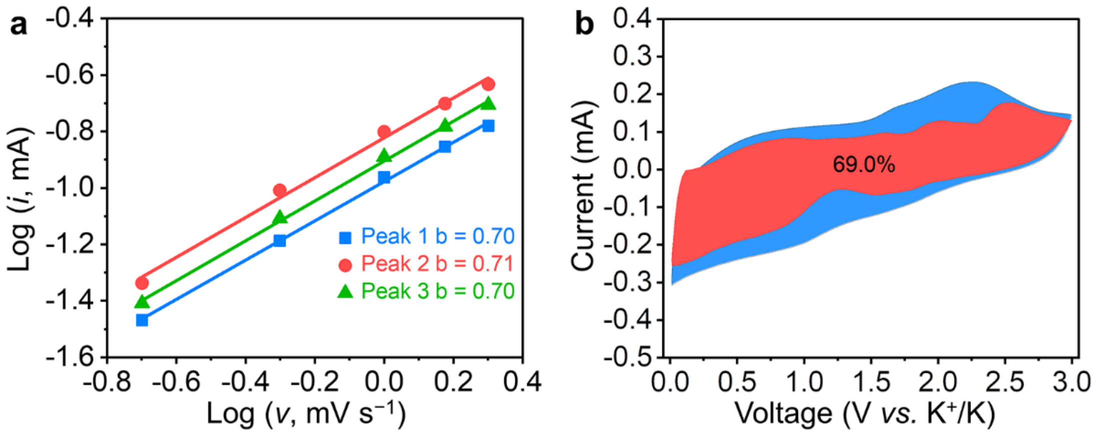

Fig. S11 (**a**) The relationship between log *i* and log *v* of the $CoTe_2$@NC@NSPCNFs electrode, where *i* is the peak current, *v* is the scan rate, and *b* is the slope of log (*i*) *vs*. log (*v*), and (**b**) the capacitive contribution (red) of the $CoTe_2$@NC@NSPCNFs electrode at a scan rate of 2.0 mV s^{-1}

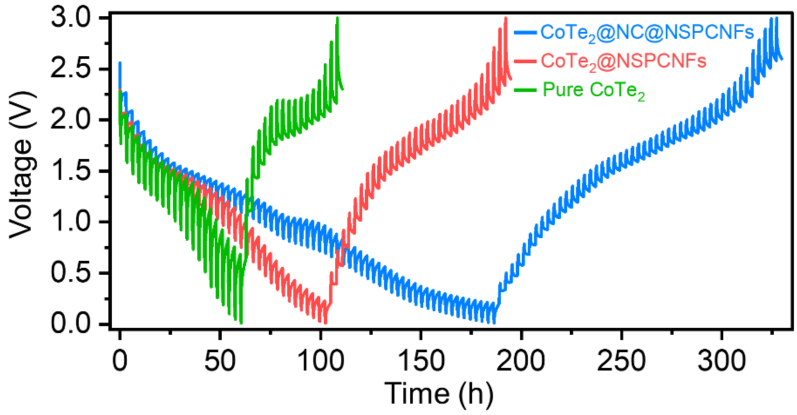

Fig. S12 Galvanostatic intermittent titration technique (GITT) curves of the $CoTe_2$@NC@NSPCNFs, $CoTe_2$@NSPCNFs, and pure $CoTe_2$ electrodes

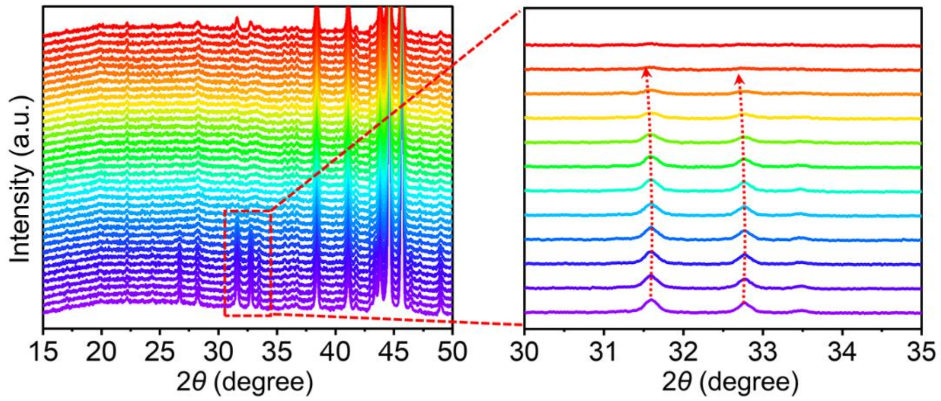

Fig. S13 *In-situ* XRD patterns of the $CoTe_2$@NC@NSPCNFs electrode during the first cycle

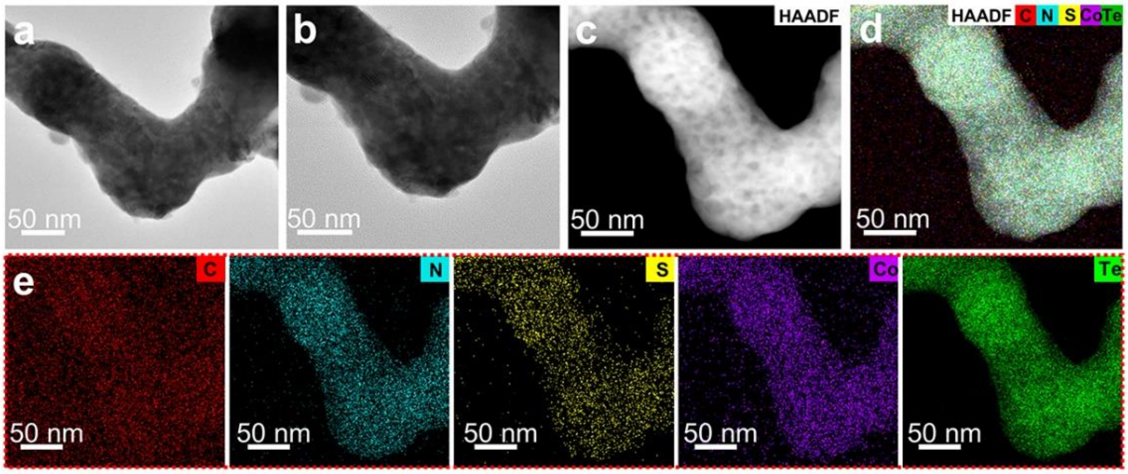

Fig. S14 (**a**, **b**) TEM and (**c**-**e**) elemental mapping images of the $CoTe_2$@NC@NSPCNFs electrode discharged to 0.01 V

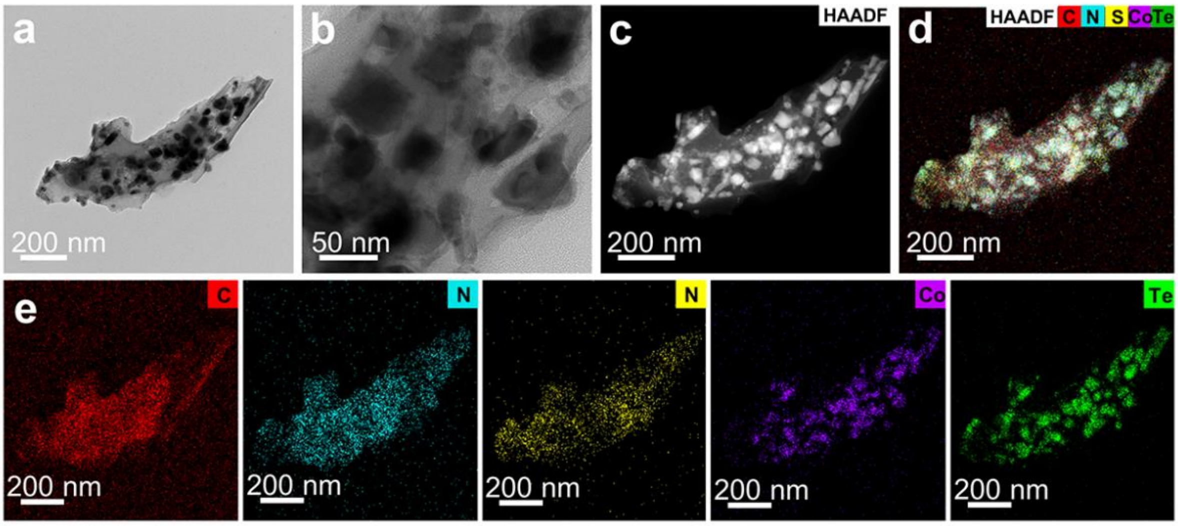

Fig. S15 (**a**, **b**) TEM and (**c**-**e**) elemental mapping images of the $CoTe_2$@NC@NSPCNFs electrode charged to 3.0 V

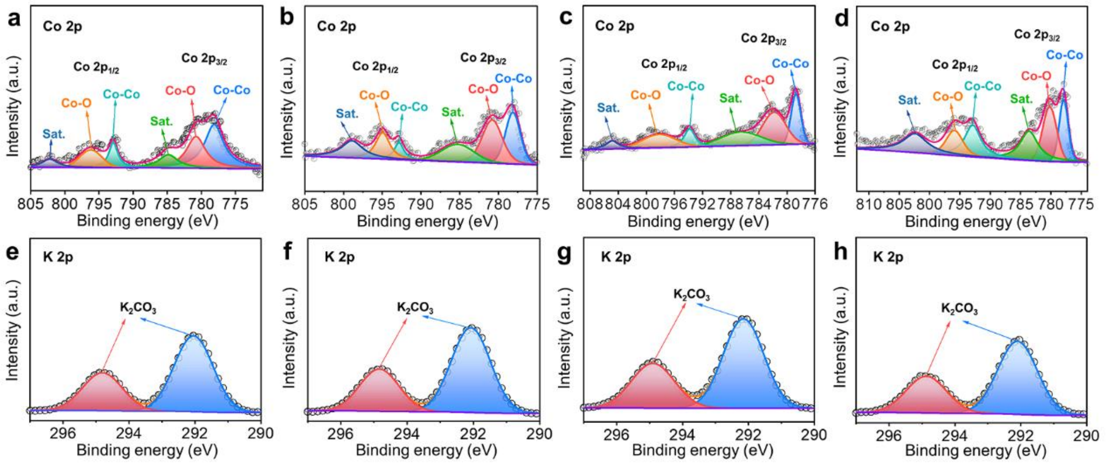

Fig. S16 XPS spectra of Co 2p and K 2p were analyzed after the initial discharged and charged states of the $CoTe_2$@NC@NSPCNFs electrode: (**a**, **e**) discharge to 0.8 V, (**b**, **f**) discharge to 0.4 V, (**c**, **g**) discharge to 0.01 V and (**d**, **h**) charge to 1.5 V

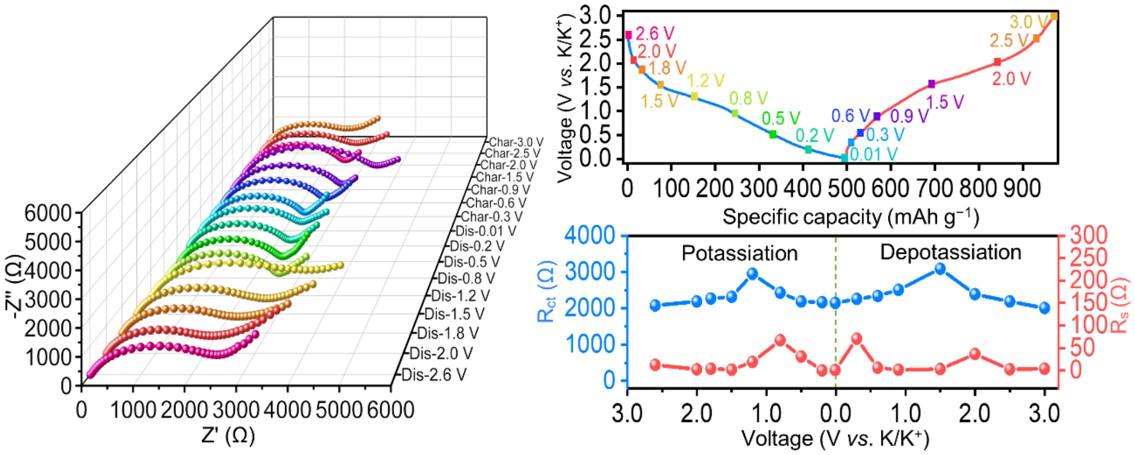

Fig. S17 *In-situ* EIS curves and the corresponding impedances of the $CoTe_2$@NSPCNFs@NC electrode during the initial cycling

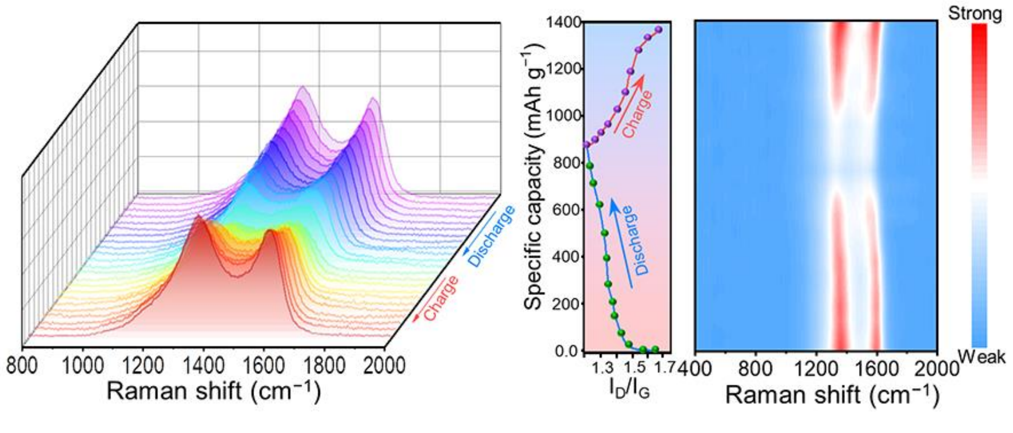

Fig. S18 *In-situ* Raman spectra and the corresponding contour plot of the $CoTe_2$@NSPCNFs@NC electrode during the initial cycling

As shown in Fig. S18, the potassium-storage behavior of the $CoTe_2$@NC@NSPCNFs electrode was further analyzed by *in-situ* Raman spectroscopy. During the discharge process, the D and G bands gradually redshift, which corresponds to the charge transfer effects after the K^+ intercalation. Notably, the intensity of the D band gradually becomes weak and the value of the I_D/I_G decreases from 1.66 to 1.21, which can be attributed to the introduction of K^+ onto the defective sites, thus reducing the optical skin depth. Impressively, the D and G bands, including the I_D/I_G value, can return to the original state during the charging process, suggesting the excellent structural stability of the $CoTe_2$@NC@NSPCNFs electrode.

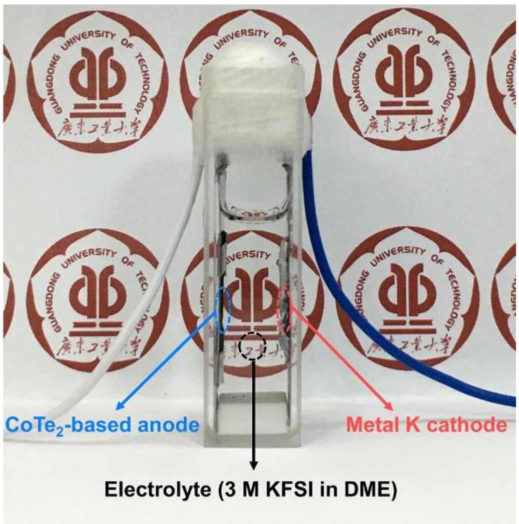

Fig. S19 The electrolytic cell for the collection of *in-situ* UV-vis absorption spectra.

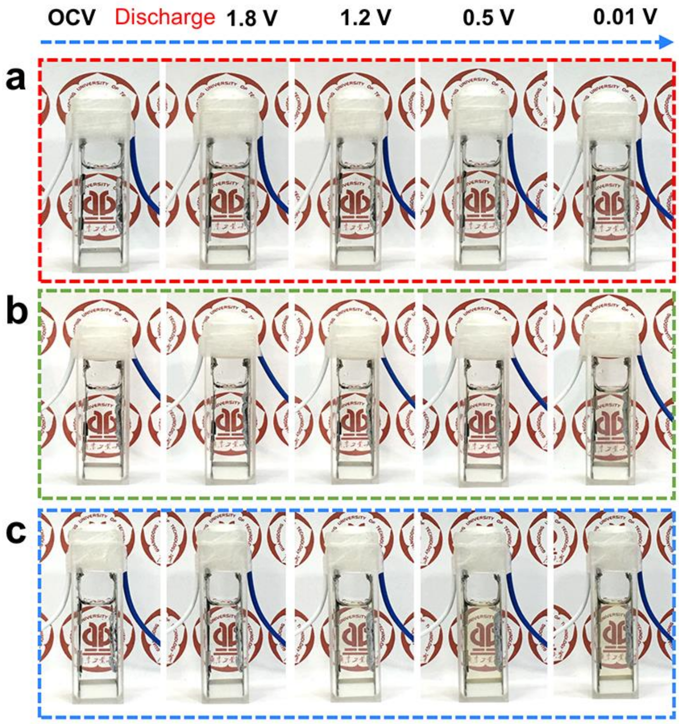

Fig. S20 The electrolytic cell visualizes the dissolution and shuttling behavior after the initial fully discharged state: (**a**) $CoTe_2$@NC@NSPCNFs, (**b**) $CoTe_2$@NSPCNFs, and (**c**) pure $CoTe_2$

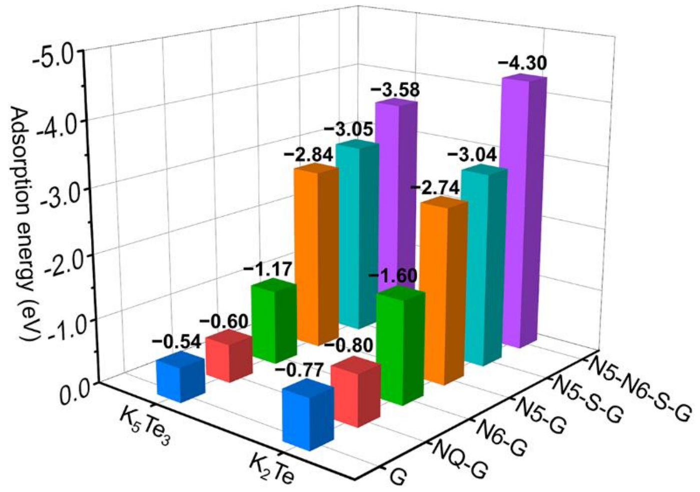

Fig. S21 The adsorption energy of K_5Te_3 and K_2Te adsorbed on G, NQ-G, N6-G, N5-G, N5-S-G, and N5-N6-S-G substrates, respectively

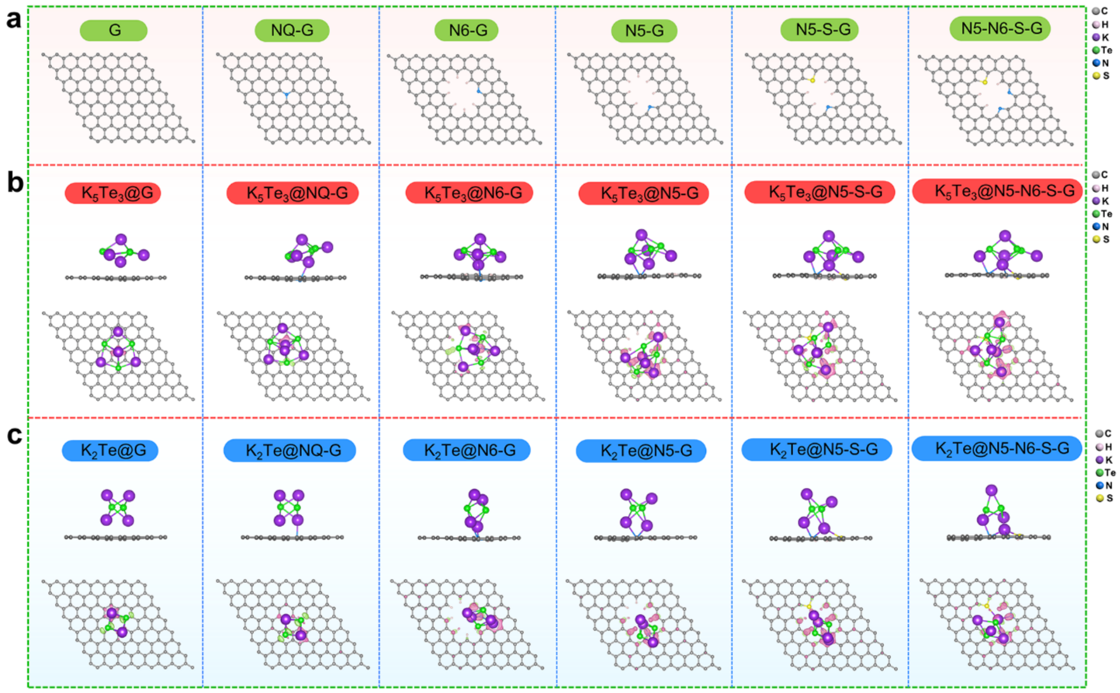

Fig. S22 (**a**) Top views of G, NQ-G, N6-G, N5-G, N5-S-G, and N5-N6-S-G carbon structure. Side views of (**b**) K_5Te_3 and (**c**) K_2Te absorbed on different graphene substrates and top views of the corresponding electron density differences. Pink and light green areas represent electron accumulation and depletion, respectively

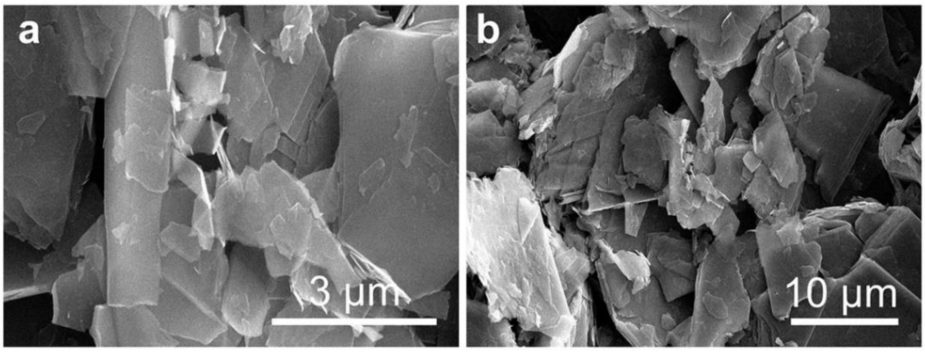

Fig. S23 SEM images of graphite

As shown in Fig. S23, the morphology of graphite is a large sheet-like structure.

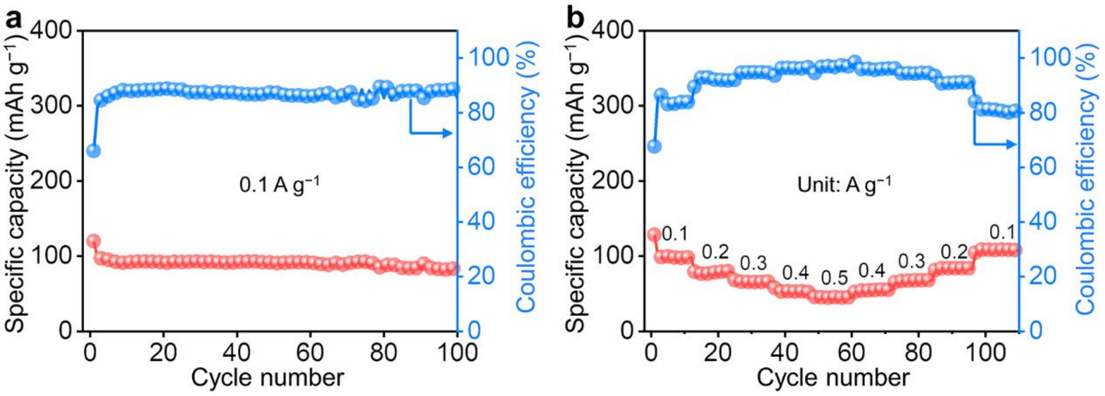

Fig. S24 The electrochemical performance of the graphite cathode with 5M KFSI/EC/DMC electrolyte: (**a**) the cycling performance at 0.1 A g^{-1}, and (**b**) the rate capability from 0.1−0.5 A g^{-1}

It can be seen from Fig. S24a that the graphite cathode exhibits a capacity of 87.4 mAh g^{-1} after 100 cycles at 0.1 A g^{-1}. Moreover, the rate capability of the graphite cathode was studied, and the results are shown in Fig. S24b. The graphite cathode exhibits high reversible capacities of 99.3, 76.2, 65.5, 57.4, and 45.0 mAh g^{-1} from 0.1 to 0.5 A g^{-1}, respectively.

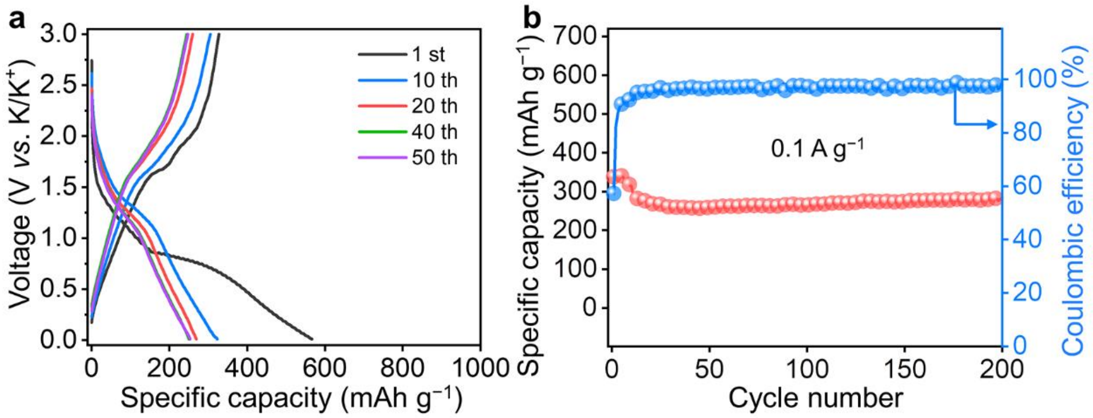

Fig. S25 (**a**) The charge-discharge profiles and (**b**) cycling performance of the $CoTe_2$@NC@NSPCNFs anode at 0.1 A g^{-1} with the electrolyte consisting of 5M KFSI in EC/DMC

As exhibited in Fig. S25, the initial discharge/charge capacities of the $CoTe_2$@NC@NSPCNFs electrode are 566.7/327.3 mAh g^{-1} with a Coulombic efficiency (CE) of 57.2%. In addition, the $CoTe_2$@NC@NSPCNFs electrode delivers a capacity of 283.8 mAh g^{-1} after 200 cycles at 0.1 A g^{-1}.

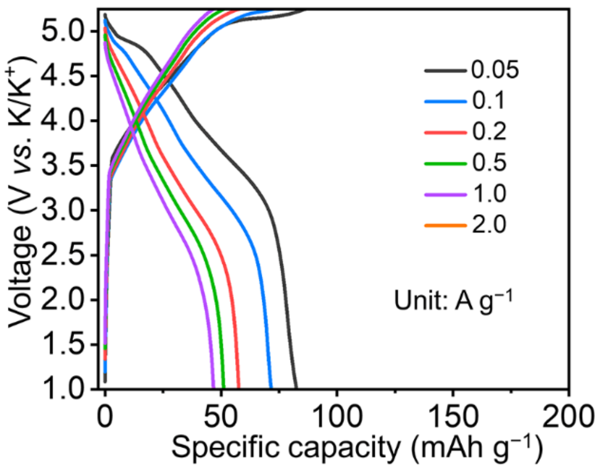

Fig. S26 Selected galvanostatic charge/discharge profiles of the $CoTe_2$@NC@NSPCNFs//graphite PDIBs at different current densities

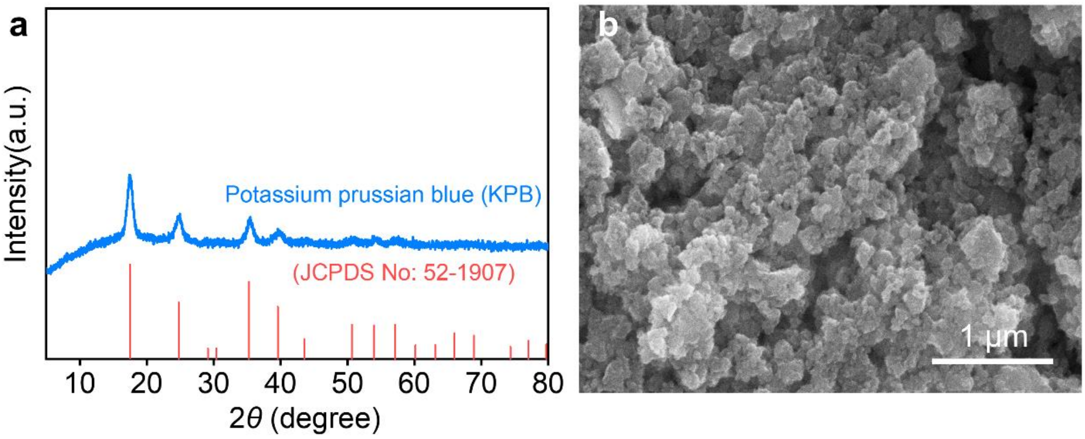

Fig. S27 (**a**) The XRD pattern and (**b**) SEM image of KPB

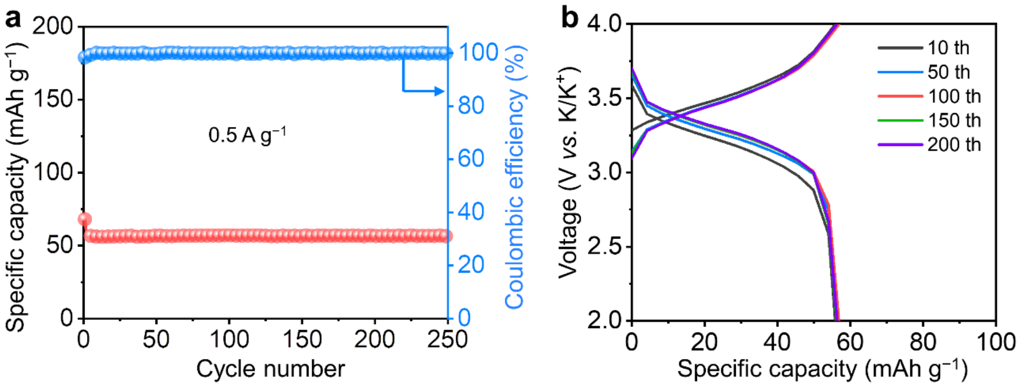

Fig. S28 (**a**) Cycling performance of KPB cathode in half cells at 0.5 A g^{-1} and (**b**) the corresponding charge/discharge profiles at different cycles

As shown in Fig. S28a, the KPB cathode delivers a stable specific capacity of 56.5 mAh g^{-1} after 250 cycles at 0.5 A g^{-1}. Furthermore, the charge and discharge voltage plateaus are located at 3.45 V and 3.28 V, respectively, indicating that the KPB electrode is an appropriate cathode for K-ion full cells (Fig. S28b).

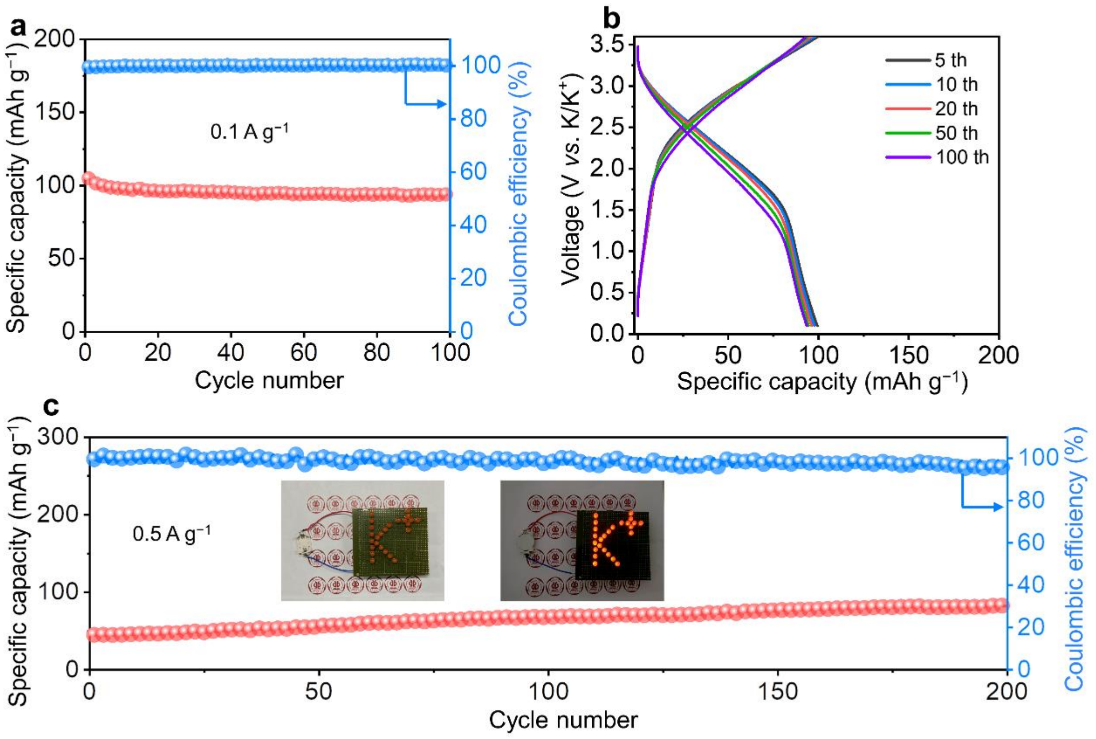

Fig. S29 Electrochemical performance of the $CoTe_2$@NC@NSPCNFs//KPB full cell: **(a)** Cycling performance at 0.1 A g^{-1}, (**b**) the corresponding charge/discharge profiles at 0.1 A g^{-1}, and (**c**) cycling performance at 0.5 A g^{-1} (Inset: Photograph of LED arrays was powered by one $CoTe_2$@NC@NSPCNFs//KPB full cell)

It can be found from Fig. S29a that the $CoTe_2$@NC@NSPCNFs//KPB full cell delivers a capacity of 93.8 mA h g^{-1} after 100 cycles at the current density of 0.1 A g^{-1}. Moreover, the average discharge plateau of $CoTe_2$@NC@NSPCNFs//KPB full cells is estimated to be 2.2 V under the voltage window of 0.1~3.6 V according to the voltage profiles in different cycles (Fig. S29b). Furthermore, the $CoTe_2$@NC@NSPCNFs//KPB full cell shows a specific capacity of 81.9 mAh g^{-1} after 200 cycles at 0.5 A g^{-1} (Fig. S29c). Impressively, the $CoTe_2$@NC@NSPCNFs//KPB full cells can powder the light emitting diode (LED) array with the label of "K$^+$", which indicates the great potential of $CoTe_2$@NC@NSPCNFs anode for practical applications.

Table S1 The density of typical transition metal tellurides

Materials	$CoTe_2$	Sb_2Te_3	SnTe	$CuTe_2$	GeTe	FeTe	ZnTe
Density (g cm^{-3})	7.92	6.5	6.48	7.1	6.14	6.25	6.34

Table S2 The element content of $CoTe_2$@NC@NSPCNFs based on XPS analysis

Element	C	N	S	O	Co	Te
Atomic content%	67.4	7.5	1.3	9.7	2.3	11.8

Table S3 Atomic contents of pyridinic-N (N-6), pyrrolic-N (N-5), and graphitic-N (N-Q) in $CoTe_2$@NC@NSPCNFs

Nitrogen type	N-6	N-5	N-Q
Atomic content (%)	43.5	43.3	13.2

Table S4 The contributed reversible capacity of each component ($CoTe_2$ and C) in the $CoTe_2$@NC@NSPCNFs composite

Current density (A g^{-1})	Cycle number	$CoTe_2$@NC@NSPCNFs (mAh g^{-1})	NC@NSPCNFs (mAh g^{-1})	Ratio (NC@NSPCNFs / $CoTe_2$@NC@NSPCNFs)
1.0	500	186.6	47.7	8.7%
2.0	500	140.5	10.6	2.6%

The specific capacities of S/N-doped porous carbon nanofibers (NC@NSPCNFs) in $CoTe_2$@NC@NSPCNFs are about 88.4 and 18.3 mAh g^{-1} after 200 and 300 cycles at 0.5 and 2.0 A g^{-1}, respectively. Based on the reversible capacity and carbon content, the capacity contribution of NC@NSPCNFs in the $CoTe_2$@NC@NSPCNFs composite was calculated. In detail, the capacity contribution ratio of NC@NSPCNFs in the $CoTe_2$@NC@NSPCNFs composite is 8.7% ($\frac{34.1\%\times 47.7}{186.6} \approx 8.7\%$) at 1.0 A g^{-1} and 2.6% ($\frac{34.1\%\times 10.6}{140.5} \approx 2.6\%$) at 2.0 A g^{-1}, respectively.

Table S5 Potassium-storage performance of the $CoTe_2$@NC@NSPCNFs electrode compared with other TMTes materials in previous reports

**Sample**	**Current density (A g^{-1})**	**Cycle number**	**Capacity (mAh g^{-1})**	**References**
$CoTe_2$@NC@NSPCNFs	0.05	50	428.9	This work
	0.1	1100	329.4	
	1.0	2000	194.5	
	2.0	3500	110.9	
$CoTe_2$-C composite	0.5	300	189.5	[S2]
TeNWs/CNTs/rGO	0.084	100	168	[S6]
$SbBiTe_3$	0.08	1000	202	[S7]
$FeTe_2$-C	0.5	500	171	[S8]
Bi_2Te_3@C	1.0	500	204	[S9]
WTe_2	0.1	10	143.3	[S10]
$MoTe_2$	0.1	50	104	[S11]

Table S6 The fitted resistances from the EIS curves of $CoTe_2$@NC@NSPCNFs electrode before and after different cycles

$CoTe_2$@NC@NSPCNFs	R_s (Ω)	R_{ct} (Ω)
Before cycling	0.79	2020
After 5 cycles	3.54	1590
After 50 cycles	2.04	1200
After 100 cycles	1.23	1180

Table S7 The fitted resistances from the EIS curves of $CoTe_2$@NSPCNFs electrode before and after different cycles

$CoTe_2$@NSPCNFs	R_s (Ω)	R_{ct} (Ω)
Before cycling	1.35	3820
After 5 cycles	0.86	2430
After 50 cycles	1.35	2660
After 100 cycles	0.4	4070

Table S8 The fitted resistances from the EIS curves of pure $CoTe_2$ electrode before and after different cycles

Pure $CoTe_2$	R_s (Ω)	R_{ct} (Ω)
Before cycling	4.31	5120
After 5 cycles	2.84	2860
After 50 cycles	3.82	7140
After 100 cycles	1.41	12000

Table S9 Comparison of the operating voltage between the $CoTe_2$@NC@NSPCNFs//graphite PDIBs and previously reported PDIBs

**PDIBs**	**The range of operating voltage (V)**	**References**
$CoTe_2$@NC@NSPCNFs//graphite	1.0 – 5.25	This work
pK_2TP//EG	0.5 – 4.0	[S12]
SnSb@PCNWs//EG	2.5 – 5.0	[S13]
PTPAn//Graphite	1.0 – 4.0	[S14]
Graphite//Graphite	3.0 – 5.0	[S15]
HPSbCNFs-70//Graphite	3.0 – 5.0	[S16]
HPNCFs//Graphite	3.0 – 5.0	[S17]
Graphite//CEI-modified graphite	3.0 – 5.0	[S18]
Graphite//Graphite	2.5 – 5.0	[S19]
N-AHCSs//EG	3.0 – 5.0	[S20]
MoS_2//Graphite	1.0 – 5.0	[S21]

Supplementary References

[S1]W. Zhang, X. Wang, K. W. Wong, W. Zhang, T. Chen et al., Rational design of embedded $CoTe_2$ nanoparticles in freestanding N-doped multichannel carbon fibers for sodium-ion batteries with ultralong cycle lifespan. ACS Appl. Mater. Interfaces **13**(29), 34134−34144 (2021). https://doi.org/10.1021/acsami.1c06794

[S2]S. Yang, G. D. Park, Y. C. Kang Conversion reaction mechanism of cobalt telluride-carbon composite microspheres synthesized by spray pyrolysis process for K-ion storage. Appl. Surf. Sci. **529**, 147140 (2020). https://doi.org/10.1016/j.apsusc.2020.147140

[S3]Q. Li, W. Zhang, J. Peng, W. Zhang, Z. Liang et al., Metal-organic framework derived ultrafine Sb@porous carbon octahedron via in situ substitution for high-performance sodium-ion batteries. ACS Nano **15**(9), 15104−15113 (2021). https://doi.org/10.1021/acsnano.1c05458

[S4]Q. Li, W. Zhang, J. Peng, D. Yu, Z. Liang et al., Nanodot-in-nanofiber structured carbon-confined Sb_2Se_3 crystallites for fast and durable sodium storage. Adv. Funct. Mater. **32**(18), 2112776 (2022). https://doi.org/10.1002/adfm.202112776

[S5]M. Sha, L. Liu, H. Zhao, Y. Lei Anode materials for potassium-ion batteries: Current status and prospects. Carbon Energy **2**(3), 350−369 (2020). https://doi.org/10.1002/cey2.57

[S6] Q. Liu, W. Deng, C.-F. Sun A potassium-tellurium battery. Energy Storage Mater. **28**, 10−16 (2020). https://doi.org/10.1016/j.ensm.2020.02.021
[S7] J. Cui, S. Yao, M. Ihsan-Ul-Haq, N. Mubarak, M. Wang et al., Rational exploration of conversion-alloying reaction based anodes for high-performance K-ion batteries. ACS Mater. Lett. **3**(4), 406−413 (2021). https://doi.org/10.1021/acsmaterialslett.0c00627 n
[S8] G. D. Park, Y. C. Kang Conversion reaction mechanism for yolk-shell-structured iron telluride-C nanospheres and exploration of their electrochemical performance as an anode material for potassium-ion batteries. Small Methods **4**(10), 2000556 (2020). https://doi.org/10.1002/smtd.202000556
[S9] J. K. Ko, J. H. Jo, H. J. Kim, J. S. Park, H. Yashiro et al., Bismuth telluride anode boosting highly reversible electrochemical activity for potassium storage. Energy Storage Mater. **43**, 411−421 (2021). https://doi.org/10.1016/j.ensm.2021.09.028
[S10] D. M. Soares, G. Singh Superior electrochemical performance of layered WTe_2 as potassium-ion battery electrode. Nanotechnol. **31**(45), 455406 (2020). https://doi.org/10.1088/1361-6528/ababcc
[S11] B. Wu, J. Luxa, E. Kovalska, M. Ivo, H. Zhou et al., Sub-millimetre scale van der waals single-crystal $MoTe_2$ for potassium storage: Electrochemical properties, and its failure and structure evolution mechanisms. Energy Storage Mater. **43**, 284−292 (2021). https://doi.org/10.1016/j.ensm.2021.09.006
[S12] A. Yu, Q. Pan, M. Zhang, D. Xie, Y. Tang Fast rate and long life potassium-ion based dual-ion battery through 3D porous organic negative electrode. Adv. Funct. Mater. **30**(24), 2001440 (2020). https://doi.org/10.1002/adfm.202001440
[S13] C. Wei, D. Gong, D. Xie, Y. Tang The free-standing alloy strategy to improve the electrochemical performance of potassium-based dual-ion batteries. ACS Energy Lett. **6**(12), 4336−4344 (2021). https://doi.org/10.1021/acsenergylett.1c02092
[S14] L. Fan, Q. Liu, Z. Xu, B. Lu An organic cathode for potassium dual-ion full battery. ACS Energy Lett. **2**(7), 1614−1620 (2017). https://doi.org/10.1021/acsenergylett.7b00378
[S15] L. Fan, Q. Liu, S. Chen, K. Lin, Z. Xu et al., Potassium-based dual ion battery with dual-graphite electrode. Small **13**(30), 1701011 (2017). https://doi.org/10.1002/smll.201701011
[S16] M. Zhang, J. Zhong, W. Kong, L. Wang, T. Wang et al., A high capacity and working voltage potassium-based dual ion batteries. Energy Environ. Mater. **4**(3), 413−420 (2020). https://doi.org/10.1002/eem2.12086
[S17] M. Zhang, M. Shoaib, H. Fei, T. Wang, J. Zhong et al., Hierarchically porous N-doped carbon fibers as a free-standing anode for high-capacity potassium-based dual-ion battery. Adv. Energy Mater. **9**(37), 1901663 (2019). https://doi.org/10.1002/aenm.201901663
[S18] Q. Wang, W. Liu, S. Wang, M. Tan, S. Luo et al., High cycling stability graphite cathode modified by artificial CEI for potassium-based dual-ion batteries. J. Alloys Compd. **918**, 165436 (2022). https://doi.org/10.1016/j.jallcom.2022.165436
[S19] K. X. Li, G. Y. Ma, D. D. Yu, W. Luo, J. X. Li et al., A high-concentrated and nonflammable electrolyte for potassium ion-based dual-graphite batteries. Nano Res. **12**, 1−8 (2023). https://doi.org/10.1007/s12274-023-5438-z
[S20] Q. Wang, S. Wang, W. Liu, D. Wang, S. Luo et al., N-doped hollow carbon spheres as a high-performance anode for potassium-based dual-ion battery. J. Energy Storage. **54**, 105285 (2022). https://doi.org/10.1016/j.est.2022.105285
[S21] A. Kotronia, K. Edström, D. Brandell, H. D. Asfaw Ternary ionogel electrolytes enable quasi-solid-state potassium dual-ion intercalation batteries. Adv. Energy Sustainability Res. **3**(1), 2100122 (2021). https://doi.org/10.1002/aesr.202100122
